# Adaptation and resistance of soil prokaryotic communities to drought intensification in old-growth forests and pastures of southwestern Amazonia

**DOI:** 10.3389/fpls.2025.1684321

**Published:** 2025-11-03

**Authors:** Elisa Díaz García, Diana Boy, Simone Kilian Salas, Alberto Andrino, Leopold Sauheitl, Anja Poehlein, Georg Guggenberger, Marcus A. Horn, Jens Boy

**Affiliations:** ^1^ Institute of Earth System Sciences, Section Soil Science, Leibniz University Hannover, Hannover, Germany; ^2^ Institute of Microbiology, Leibniz University Hannover, Hannover, Germany; ^3^ iES Landau, RPTU Kaiserslautern-Landau, Landau in der Pfalz, Germany; ^4^ Genomic and Applied Microbiology & Göttingen Genomics Laboratory, Georg-August University, Göttingen, Germany

**Keywords:** Drying-rewetting, land use, root exudates, soil prokaryotic community, soil respiration

## Abstract

**Introduction:**

Climate change is predicted to intensify droughts in tropical regions. However, the extent to which drought intensification and the subsequent changes in root exudate (RE) composition reshape soil prokaryotic communities (SPC) remains poorly understood.

**Methods:**

We conducted a 69-day incubation to determine the effects of repeated exposure to severe drought and RE application on the SPC activity and structure in soils under old-growth forests and pastures from southwestern Amazonia. At the beginning of each cycle, microcosms received either artificial RE solution or sterile water; following drying, microcosms were either kept at 30% water holding capacity (WHC) for 18 days, representing the regional WHC in the dry season, or at 5% WHC, simulating severe drought.

**Results:**

Drought intensity and RE availability were the primary drivers of changes in SPC composition and activity. The lowest prokaryotic diversity values were observed in the severe drought treatment with +RE addition for both land-uses. After wetting, +RE microcosms showed higher SPC activity due to the utilization of the supplemented REs. Carbon availability interacted with land-use specific characteristics and partially buffered drought effects on SPC composition in pastures. The SPCs from both land-uses were well-adapted to regional drought conditions. However, repeated severe drought caused significant community shifts towards dominance of a few drought-resistant families.

**Discussion:**

Intensifying droughts can reduce prokaryotic diversity and reassemble tropical soil communities toward drought-tolerant taxa, with RE inputs amplifying pos-wetting activity yet exacerbating diversity losses under severe stress. Such changes may compromise ecosystem stability and soil functions under future precipitation regimes.

## 1 Introduction

The Amazon Basin is increasingly threatened by deforestation, which leads to accelerated climate change, disruption of the regional water cycle, loss of its remarkable biodiversity, and decline in the value of the ecosystem services in the region (Davidson et al., 2012; Nobre et al., 2016). Cattle ranching is the main driver of deforestation in the Amazon, accounting for approximately 80% of the forest loss, mainly through slash-and-burn clearing (Foley et al., 2007). The removal of forest cover exposes the pasture soil to direct and constant wind and water erosion, which contributes to the gradual loss of the nutrient-rich topsoil that is essential for plant growth (Martínez and Zinck, 2004; Veldkamp et al., 2020). The cumulative effects of cattle grazing and trampling intensifies nutrient loss and soil compaction. The degradation of the pasture soils is evident as they become less productive over time (Davidson et al., 2012; Nobre et al., 2016). Rainforests adjacent to pastures are additionally at a higher risk of degradation due to increased exposure to anthropogenic activities (Foley et al., 2007; Nobre et al., 2016), e.g. fires or agricultural management practices. These activities disrupt the ecological balance of the forest, perpetuate the cycle of deforestation, and decrease the ecosystem resilience (Davidson et al., 2012). In fact, a reduction of 20-25% forest cover in the Amazon Basin could trigger a rapid land conversion to savanna-like landscapes, particularly in regions that are naturally close to the threshold of precipitation needed for the development of a rainforest, such as the southwestern Amazon (Lovejoy and Nobre, 2018).

Along with deforestation, additional stressors such as climate change are pushing the southwestern Amazon forest towards a savanna state, as evidenced by the increasing frequency and severity of droughts in recent decades (Lovejoy and Nobre, 2018). Severe droughts in 2005 and 2010 have been linked to rising Pacific sea surface temperatures (Lewis et al., 2011; Zeng et al., 2008), while intense drought conditions in 2015 and 2023 were associated with strong El Niño events (Espinoza et al., 2024; Jiménez-Muñoz et al., 2016), emphasizing the region’s growing exposure to climate extremes. Changes in drought patterns are projected to intensify in the future due to the acceleration of climate change and deforestation rates (Baldrian et al., 2023; Davidson et al., 2012). The most direct effects of droughts are a decrease in soil moisture levels and water availability for plants, which is often less severe in forest soils. These ecosystems have deep-rooted trees and stable microclimates that buffer temperature fluctuations (Davidson et al., 2000; Nepstad et al., 1996), while maintaining higher soil moisture and more stable conditions through the year compared to pasture soils. Nevertheless, under prolonged drought stress both land-uses will face ecological impacts. Low soil water availability directly affects the soil microbial community (Baldrian et al., 2023; Schimel, 2018), which includes prokaryotes (bacteria and archaea) and fungi. Microorganisms require water for their metabolic processes, therefore, drought conditions can cause a decline in microbial biomass and microbial activity (Baldrian et al., 2023; Naylor and Coleman-Derr, 2018), and consequently, soil respiration rates (CO_2_ emissions) are also reduced (Li et al., 2018; Schimel, 2018). During the drought period this reduction in CO_2_ emissions could be perceived as increased carbon sequestration. However, it has been demonstrated that the rewetting of dry soils results in a pulse of CO_2_ emissions, known as the Birch effect, which can be many times greater than the basal respiration rates of the soil (Naylor and Coleman-Derr, 2018; Schimel, 2018). Moreover, prolonged and intense droughts can also reduce soil prokaryotic diversity (Bouskill et al., 2013), thus decreasing the functional redundancy of the soil microbial community (Bérard et al., 2015). Previous studies have shown that under low soil water content the soil prokaryotic community (SPC) often starts to adapt, potentially becoming dominated by drought-resistant species, such as those with thick cell walls, which are generally found in gram-positive prokaryotes (Naylor and Coleman-Derr, 2018; Schimel et al., 2007) or species capable of producing spores (Bérard et al., 2015; Naylor and Coleman-Derr, 2018; Schimel, 2018). Compositional and structural shifts decrease the functional capabilities of the SPC, affecting its resistance to future environmental challenges (Bouskill et al., 2013; Mendes et al., 2015).

As soils dry, plants also adapt their physiological processes to cope with the stress, affecting the surrounding SPC. As a survival strategy, plants can modify the quantity and composition of their root exudates (Maurer et al., 2021; Naylor and Coleman-Derr, 2018; Vives-Peris et al., 2020). Root exudates (RE) are organic compounds permanently secreted by plant roots into the surrounding soil and include a mix of primary metabolites (saccharides, amino acids, organic acids, etc.), secondary metabolites (flavonoids, auxins, etc.) and high-molecular weight compounds as proteins (Badri and Vivanco, 2009; Vives-Peris et al., 2020). In the nutrient-poor soils of the Amazon region (Vitousek, 1984), REs are of utmost importance for sustaining plant and soil microbial communities, as some are able to change the soil pH and mobilize plant-growth limiting nutrients, such as phosphorus (Almeida et al., 2020; Aoki et al., 2012). Microorganisms can consume REs secreted by plants (Huang et al., 2014; Luo et al., 2014), while simultaneously SPCs decomposing soil organic matter and releasing additional nutrients into the environment. Nutrient availability and nutrient uptake are enhanced by this symbiotic relationship, promoting the growth and activity of plants and microorganisms (Badri and Vivanco, 2009; Bai et al., 2022; Huang et al., 2014), and improve their resistance to stresses (Bai et al., 2022; Vives-Peris et al., 2020).

Despite of their relevance for many ecosystem processes, little scientific attention has been given to studies on microbiomes in the presence or absence of RE. The intrinsic variability of biotic (vegetation composition, shifts due to anthropogenic use, etc.) and abiotic factors (temperature, rainfall, wind, etc.) presents critical challenges for understanding the impact of drought and RE secretion by plants on the SPC (Bai et al., 2022; Huang et al., 2014; Maurer et al., 2021; Vives-Peris et al., 2020). In tropical ecosystems the challenge is even greater due to the rich plant diversity and the inaccessibility for researchers to study sites. Nevertheless, studies on greenhouse gases have reported that drought-stressed plants increase the secretion of certain organic acids such as oxalic acid (Almeida et al., 2020; Gargallo-Garriga et al., 2018; Naylor and Coleman-Derr, 2018), commonly found in root exudates (Jones et al., 2003). Oxalic acid can particularly enhance phosphorus (P) solubilization (Almeida et al., 2020; Ström et al., 2002) and aluminum detoxification of plants (Graşz et al., 2009) in highly weathered, acidic soils typical of the Amazonia. Moreover, experimental studies have shown that during drought conditions, the REs contain a higher concentration of stress-related compounds, such as abscisic acid, flavonoids (Gargallo-Garriga et al., 2018), and primary metabolites associated with osmotic adjustment (Lopes et al., 2022; Schimel et al., 2007), such as saccharides (Chen et al., 2022; Gargallo-Garriga et al., 2018) and specific amino acids, e.g. aspartate and leucine (Gargallo-Garriga et al., 2018), which attract microorganisms (Huang et al., 2014; Vives-Peris et al., 2020). The analyses of complex interactions are highly limited due to multiple factors acting simultaneously, the challenges in isolating and controlling specific variables, and the difficulty in determining both their individual and cumulative effects, particularly in systems exhibiting non-linear behaviors.

In this complex context, incubation experiments in microcosms offer the additional advantage of controlled conditions, allowing researchers to isolate and focus on specific variables. Furthermore, these experiments are easily reproducible, yielding more uniform results. However, as closed systems, they do not allow interactions with the surrounding environment, limiting their ecological realism and applicability to natural settings. For this reason, the addition of artificial REs into microcosms enhances the simulation of more realistic scenarios (Baumert et al., 2018; Steinauer et al., 2016). Indeed, the SPC can be influenced not only by drought intensification and shifts in root exudate composition (Chen et al., 2022; Naylor and Coleman-Derr, 2018; Preece and Peñuelas, 2016), but also by land use (Danielson and Rodrigues, 2022; Paes da Costa et al., 2022; Rodrigues et al., 2013). These changes can have lasting effects on the SPC, even after the drought stress has ceased (Bouskill et al., 2013; Canarini et al., 2021; Moreno et al., 2019) and/or land use has changed (Moreno et al., 2019; Paula et al., 2014).

The main aim of this study was to simulate drought intensification and assess its impact on SPC activity, diversity, and composition in tropical regions. To establish a realistic baseline of pre-acclimatized microbial community, soils were collected at the end of the dry season. Within this context, our first hypothesis is that drought intensification reduces soil microbial activity and diversity, and selects for similar drought-tolerant taxa in both ecosystems, but with pasture SPCs exhibiting greater resistance to drought fluctuations compared to those in old-growth forests. To better simulate natural conditions, we included artificial root exudate treatments. We further hypothesized that the addition of artificial REs during drought stress promotes microbial activity and selects for drought-tolerant prokaryotic species capable of rapidly utilizing labile carbon substrates.

To test these hypotheses, we assessed the resistance of SPCs to reference (30% WHC) and severe (5% WHC) drought stress during an incubation experiment with three drying-rewetting cycles using soils from old-growth forests and active pastures in the southwestern Amazon Basin. Moreover, we simulated the effect of plant RE on shaping the SPC of old-growth forests and active pastures during drought. For this, we supplemented the incubation units with a simplified artificial RE solution composed of an amino acid (leucine), a disaccharide (trehalose) and an organic acid (oxalic acid). Leucine and trehalose are known to act as osmoprotectants (Gargallo-Garriga et al., 2018; Lopes et al., 2022), helping cells maintaining their turgor and reducing their water loss. Moreover, leucine and trehalose provide to the SPC exogenous nutrient sources that are easily assimilable, with leucine acting as a nitrogen and carbon source, and trehalose as a carbon source (Lopes et al., 2022). While oxalic acid is primarily recognized for its potential to mobilize P (Ström et al., 2002), it can also be utilized by certain soil microorganisms as a carbon source (Hervé et al., 2016), although it is generally less readily assimilable compared to leucine and trehalose. We measured the CO_2_ emissions as an indirect indicator of microbial activity during the incubation period, and used 16S rRNA gene amplicon sequencing to evaluate SPCs responses in soils under the two contrasting land use types old-growth forest and pasture.

## 2 Materials and methods

### 2.1 Study site

The soils were collected at a depth of 15 cm in the Madre de Dios region of the Peruvian Amazon at an altitude of 210–250 meters above sea level (a.s.l.). Three active pasture plots (PA) and three old-growth forest plots (FO) were selected for this study. An old-growth forest is defined in our study as a forest which has had minimal human intervention and remained almost undisturbed (Wirth et al., 2009), as completely untouched or uninfluenced forests are almost non-existent or barely accessible. All active pastures for cattle ranching were located close to old-growth forests to assure comparable abiotic environmental conditions. Pastures were established more than 30 years ago after logging and burning the previous vegetation, followed by the removal of any remaining stumps. Pastures were planted with introduced grasses, such as Panicum maximum and Brachiaria brizantha, both C4 plant species. All sampling sites were located 15–80 km away from the closest city, Puerto Maldonado. Two of the plots were located close to the Interoceanic highway that connects Puerto Maldonado with Cusco (PA1 a 30 years old pasture: 12°43’S-69°29’W, FO1: 12°53’S-69°46’W). Two sites were located in the buffer zone of the Tambopata National Reserve (PA2 a 10 years old pasture: 12°44’S-69°21’W, FO2: 12°56’S-69°31’W) and the last two sites were 800 m apart, inside the Tambopata National Reserve (FO3 and PA3 a 50 years old pasture: 12°42’S-69°09’W).

The climate in the region is humid tropical (Am) according to the Köppen Climate Classification. Rainfall is seasonal, with a dry season from June to September, an annual precipitation of 2221 mm, an average temperature of 25.4 °C, and an average relative humidity of approximately 75%. In the region, the soils are mostly Acrisols and Ferralsols (IUSS Working Group WRB, 2022) with low pH and generally low nutrient content.

Soils were collected at the end of the dry season (September 2021) to capture a microbial community pre-acclimatized to seasonal drought. Samples under pasture were taken in the center of the pasture area, around 40–300 m away from old-growth forest edges. After digging a 40 cm deep and 1 m long soil profile, 400 g of soil were taken at a depth of 15 cm along the full width of the soil profiles to obtain a composite sample. Fresh soil used for the incubation and microbial analysis was stored at 4°C in sterile tubes prior to the incubation. Soils for physicochemical analysis were sieved at 2 mm and stored air-dry until further analyses. Visible plant material was removed in the field. Moreover, a 100 cm^3^ undisturbed soil core was taken at a depth of 15 cm for bulk density analyses.

### 2.2 Experimental design

Ten grams of fresh soil (oven dry mass equivalent) were placed into 50mL autoclaved glass incubation units under sterile laboratory conditions. The experiment comprised a total of 84 incubation units (14 per plot): 42 units containing soil from the three forest plots and 42 from the three pasture plots (**Supplementary Table S1**). The diameter of the incubation units was 5 cm. At that point, the incubation units were sealed with a bromine-butyl-rubber stopper and crimped with an aluminum cap (Chroma Globe GbR, Kreuznau, Germany). UltraPure™, DNase/RNase-free distilled water (Invitrogen™) was added to achieve a water content equivalent to 30% water holding capacity (WHC) in all incubation units. Then, the incubation units were pre-incubated at 20 °C in the dark for 7 days.

**Figure 1:**
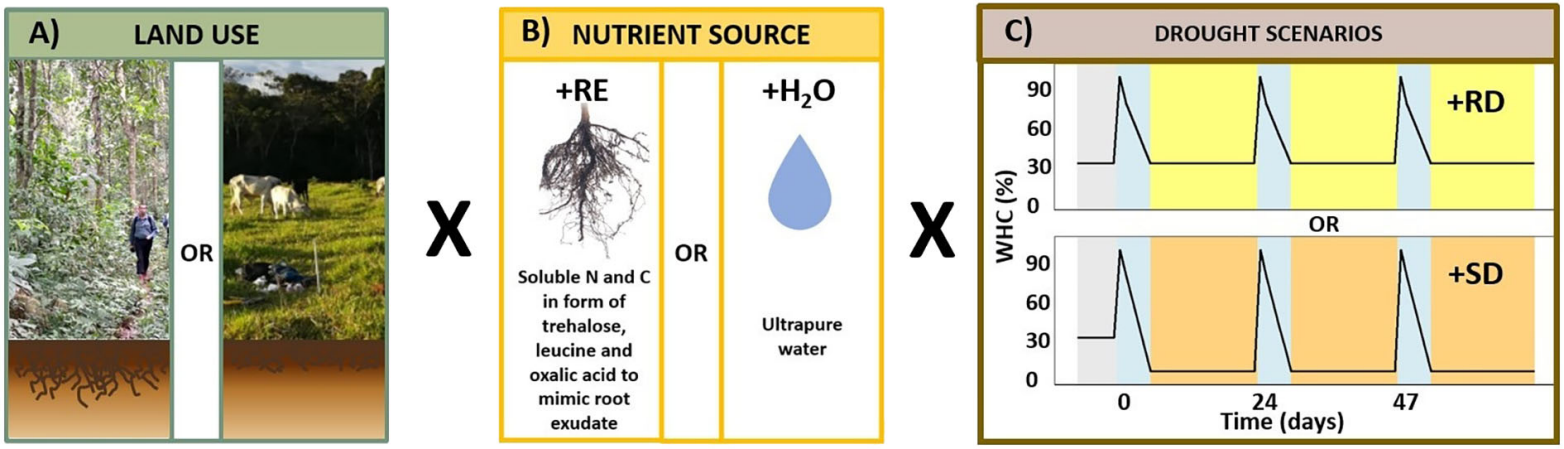
Laboratory experimental setup. **(A)** Land use type: the incubation included soils from 3 old-growth forests and 3 pastures (15 cm depth). **(B)** Nutrient source: the treatments included an artificial root exudate solution (+RE) and a baseline treatment including UltraPure™ water (+H_2_O), in which only endogenous nutrient sources were available. The +RE treatments consisted of 15 incubation units per drought scenario from 3 different sites (5 replicates per site). The +H_2_O consisted of 6 incubation units from 3 different sites (2 replicates per sites). **(C)** Drying rewetting scheme for the incubation experiment (69 days): after 7 days of pre-incubation at 30% WHC, the experiment started with the addition of the artificial RE solution or UltraPure™ water until 95% WHC, then the soils were slowly dried for a period of 5 days until the targeted WHC was reached (+RD: 30% or +SD: 5% WHC). The incubation units were stored in the dark at 20 °C for 18 days. The cycles were repeated 3 times, each lasting 23 days. Panel **(B)** root photo: Science Stock Photos, CC BY 4.0. Cropped.

After the pre-incubation phase (30% WHC), the soil samples were further incubated in the dark at 20 °C and exposed to 3 drying-rewetting cycles over 69 days with three cycles of 23 days each (**Figure 1**). Each cycle started with the wetting of the soil in the incubation units to 95% WHC using 1) UltraPure™ distilled water (+H_2_O treatment) as a baseline treatment or 2) a sterile artificial root exudate solution (+RE treatment) as a carbon and nitrogen source.

The simplified artificial RE solution consisted of a mix of three primary metabolites: sugar, amino acid, and organic acid (Baumert et al., 2018). The carbon composition of the solution was 50% from trehalose, 30% from leucine, and 20% from oxalic acid. The specific content is in line with the relative proportion of sugars, amino acids and organic acids detailed in the summary on wheat (C3 plant) published by Warren (2015). The leucine content was increased by 10% compared to Warren (2015), at the expense of a 10% decrease in organic acids, to account for other organic N sources present in the natural REs (Warren, 2015). The primary metabolites were added in reagent-grade form within the range of RE carbon fluxes in tropical evergreen forests (Chari et al., 2024). Our approach was conservative, and we used the minimum value within the reported fluxes, namely 0.14 kg C m^-2^ y^-1^ (i.e., 0.008 g C incubation unit^-1^ cycle^-1^). The three compounds were dissolved in UltraPure™ distilled water, filtered through 0.2 μm PTFE filters (Sartorius Stedim Biotech) to ensure sterility, and applied to the pre-treated soils in solution at a final concentration of 0.012 mmol RE g^-1^ dry soil (pH= 5.35 ± 0.15) to reach 95% WHC. C and N content of the solution was measured using a LiquiTOC analyzer (Elementar Analysensysteme GmbH, Germany) after its acidification with 50 μL of 32% HCl.

After wetting the soils (+RE or +H_2_O treatments), the soils were dried in steps of 75% – 50% – 30% WHC or 75% – 50% – 5% WHC, respectively, over a period of 5 days using a constant flow of sterile filtered synthetic air (80% N_2_ and 20% O_2_, Linde PLC). The last step of each cycle consisted of an 18-day drought period: half of the soils were dried to 30% WHC [13.33% (w/w)], a reference drought (+RD) to simulate conditions representative of soil water content in the southwestern Amazon rainforest during the dry season (Fernandes et al., 2002) and the other half of the incubation units were dried to 5% WHC to simulate a severe drought (+SD) (Dacal et al., 2022; Shi et al., 2015). Therefore, we had 4 different treatments per land use (**Supplementary Table S1**). The matric potential was calculated for each WHC step for both land uses (**Supplementary Tables S2**, **S3**).

In order to maintain optimal conditions within the incubation units and to prevent the accumulation of carbon dioxide (CO_2_) in the headspace, the air within the incubation units was renewed twice during the dry period by flushing the air in the headspace with synthetic air (80% N_2_ and 20% O_2_, Linde PLC). At the end of the incubation period, 0.75 g of soil were harvested under sterile conditions for DNA extraction, followed by 16S rRNA gene amplicon sequencing. The fractions were immediately frozen at -20 °C. Part of the remaining soil was dried and milled to determine the final C and N content in each incubation unit using a vario Isotope cube elemental analyzer (Elementar Analysensysteme GmbH, Hanau, Germany).

### 2.3 Soil physicochemical analysis

To conduct soil physicochemical analysis, one third of the soil from each field location was oven dried at 45 °C until a constant weight was achieved. Soil actual pH was measured in the supernatant suspension of a 1:2.5 soil to double deionized water mixture using a pH meter. Soil texture was determined for three repetitions of each soil using the pipette method for particle size analyses (Köhn, 1928; Robinson, 1922), with 0.05 M Na_4_P_2_O_7_ used as a dispersing agent.

Soil samples were milled to determine the C and N content along with δ^13^C and δ^15^N stable isotope ratios, using a vario Isotope cube (Elementar Analysensysteme GmbH, Hanau, Germany) elemental analyzer coupled to a presisION (IsoPrime Ltd, Cheadle Hulme, UK) stable isotope ratio mass spectrometer (EA-IRMS) via a continuous flow inlet using helium (99.999% purity; Linde, Munich, Germany) as the carrier gas. The δ^13^C values were corrected using Vienna Pee Dee Belemnite (VPDB) (R=0.011182) referenced standards (caffeine – IAEA-600 and cellulose – IEAE-CH-3) and the δ^15^N values were corrected using N_2_ (R=0.0036764) referenced standards (caffeine – IAEA-600; NH_4_SO_4_ – IAEA-N-1 and NH_4_SO_4_ IAEA-N-2).

Exchangeable cations and cation exchange capacity (CEC) were determined using 0.1 M barium chloride extraction (Hendershot and Duquette, 1986). Plant available nutrients were determined using the Mehlich III method (Mehlich, 1984). Measurements in all solutions (CEC, Mehlich III) were conducted using inductively coupled plasma-optical emission spectrometry (ICP-OES, Varian-725 ES, Varian Inc., Palo Alto, USA). Standard solutions were prepared from 1000 mg L^-1^ single element solution (Fisher Scientific, Loughborough, UK).

Soil water content and bulk density were determined using the undisturbed samples extracted with a 100 cm^3^ soil core. The samples were dried in an oven at 105°C for 7 days. Soil matric potential was calculated from water retention curves based on pedotransfer functions from Tomasella and Hodnett (1998) and Van Genuchten parameters were calculated accordingly to fit the pf curves, which is an effective approach for unsaturated soils but loses reliability in extreme dryness.

### 2.4 Measurement of carbon dioxide emission rates

Throughout the incubation, soil respiration was measured at 15 individual time-points for each incubation unit, corresponding to key phases of the three drying-rewetting cycles. Sampling for each cycle was conducted as follows: one day before each drying step (before drying to 75%, 50%, and 30% or 5% WHC) and twice during the dry period. This resulted in five gas samplings per cycle, on the following days: Cycle 1: Days 1, 2, 4, 6, and 23; Cycle 2: Days 24, 25, 26, 28, and 46; Cycle 3: Days 47, 48, 49, 52, and 69. Before the samplings, the incubation units were flushed with synthetic air (80% N_2_ and 20% O_2_, Linde PLC) to eliminate existing CO_2_. Then the samples were incubated at 20 °C in the dark for either 2 h during drying steps or 24 h during the dry periods. At each measurement time-point, 15 mL of gas were sampled from the headspace of each incubation unit using a gastight sterile 20 mL syringe with a Luer-Lock tip. A three-way Luer-Lock connector (B Braun AG, Melsungen, Germany) was attached between the syringe and the 0.8 mm disposable needle (Sterican G21, 40 mm, B. Braun AG, Melsungen, Germany). The gas samples were transferred to pre-evacuated 12 mL Exetainer^®^ vials, which were sealed with septum caps (IVAVW101, IVA Analysentechnik e.K., Meerbusch, Germany). Afterwards, the gas phase was replenished in each incubation unit to avoid low pressure using synthetic air. To account for any background variation in CO_2_ efflux, blanks were run alongside the soil samples, which involved measuring the signal from 4 sealed vessels containing 10 g of acid-washed sand. All gas samples were analyzed with a custom-tailored gas chromatography system, using an Agilent HP 7890B GC (Bad Camberg, Germany) as basis, as described in detail by Surey et al. (2020).

To calculate the soil respiration rate (μg CO_2_ g^-1^ dry soil day^-1^), the average CO_2_ flux from the blanks was subtracted from the CO_2_ flux from each incubation unit at the corresponding time-point measurement, the value was divided by the duration of the sampling interval and normalized per gram of dry soil. Cumulative CO_2_-C flux (μg C g^−1^ dry soil) at the end of the incubation experiment was estimated as the sum of the daily CO_2_-C emitted. The concentrations of CO_2_-C released on the days between the 15 measurement points were calculated using a cubic spline interpolation applied to the measured data to enable the calculation of cumulative CO_2_-flux (Bischoff et al., 2017).

### 2.5 Soil DNA Extraction, PCR amplification, sequencing and raw data analysis

For each microcosm, three 250 mg soil subsamples were processed in parallel using the DNeasy PowerSoil Pro Kit (Qiagen, Hilden, Germany). The lysates obtained after bead beating and inhibitor removal were combined and purified on a single spin column. After the DNA extraction, the DNA was clean and concentrated using the DNA Clean & Concentrator-5 (Zymo Research, Irvine, CA, USA), according to the manufacturers’ instructions. The V4 region of the 16S rRNA gene was amplified using the universal primers 515F: 5’-GTG CCA GCM GCC GCG GTA A-3’ (Parada et al., 2016) and 806R: 5’-GGA CTA CNN GGG TAT CTA AT -3’ (Apprill et al., 2015). Both forward and reverse primers contained overhang adapters (5’-TCGTCGGCAGCGTCA GATGTGTATAAGAGACAG-515F primer, 5’-GTCTCGTGGGCTCGGAGATGTGTATAAGAGACAG-806R primer) for compatibility with the Illumina index and sequencing adapters. Every 20 μl PCR reaction contained 2 μl diluted DNA (144 ± 51 ng DNA μL^-1^), 10 μl KAPA Hifi HotStart Polymerase (Roche Molecular Systems, Basel, Switzerland), 1 μl 1% BSA, 1 μl of forward and reverse primers (10 mM), and 5 μl UltraPure, DNase/RNase-free distilled water (Invitrogen™). Thermal cycling consisted of initial denaturation at 95 °C for 6 min, followed by 35 cycles of 95 °C for 20 s, 55 °C for 15 s, 72 °C for 10 s and finally 72 °C for 60 s using a thermal cycler (Bioer LifeTouch, Hangzhou, China). The PCR products were purified using the GeneRead Size Selection Kit (Qiagen, Hilden, Germany). Negative controls were included for both DNA extraction (no DNA template added) and 16S rRNA gene PCR to test for contamination, but no noticeable DNA contamination was observed. The concentration of DNA in the amplicons was measured using a micro volume spectrophotometer (NanoDrop Lite, Thermo Fisher Scientific, Waltham, MA, USA). Sequencing was conducted at the Illumina MiSeq platform using equipment from Illumina, San Diego, CA, USA. Dual indexing and MiSeq reagent kit v3 (600 cycles) were used as advised by the manufacturer.

### 2.6 16S rRNA gene analysis

Raw sequences were de-multiplexed and quality control was done using QIIME2 (Bolyen et al., 2019). The common method was used to remove remaining chimeric and low-quality sequences. In short, we inspected the read quality profiles, trimmed off low-quality nucleotides, filtered for error rates and dereplicated all identical sequences into unique sequences. We then merged the forward and reverse reads, created the sequence table and removed chimeras. Differences in single-nucleotide level were fixed using amplicon sequence variants (ASV) leading to a better resolution of the sequencing region (Callahan et al., 2017) compared to OTU-clustering. The taxonomic affiliation of the sequences was executed using the reference dataset of the Silva Version 138.1 (Quast et al., 2013).

### 2.7 Statistical and bioinformatics analyses

All statistical analyses were performed using R version 4.3.3 (R Core Team, 2022) and utilizing the packages *FSA* v.0.9.5 (Ogle et al., 2023), *microbiomeMarker* v.1.8.0 (Cao et al., 2022), *pairwiseAdonis* v.0.4.1 (Martinez Arbizu, 2020), *phyloseq* v.1.46.0 (McMurdie and Holmes, 2013), *plyr* v.1.8.9 (Wickham, 2011), *tidyverse* v.2.0.0 (Wickham et al., 2019), and *vegan* v.2.6-8 (Oksanen et al., 2024). A filtering step to remove low-abundant ASV was used, ASVs that occurred less than 3 times in at least 10% of the samples were removed using the package *phyloseq*. 16S rRNA ASVs data were normalized using total sum scaling. Alpha diversity indices and relative abundance were computed using *phyloseq*. All data were tested by Shapiro-Wilk test to check data normality and Levene’s test for homogeneity of variance. To assess the differences in CO_2_ emission, alpha diversity (Shannon index, Simpson index, species richness and Chao1 index), evenness (calculated as Shannon index x ln (species richness)^-1^) and relative abundance across the different treatments, data following a normal distribution was tested by analysis of variance (ANOVA), and in case of significance, followed by Tukey test. All significance levels were set at p < 0.05. For data that did not follow a normal distribution, a non-parametric Kruskal-Wallis test was performed in case of significance, followed by Dunn’s test.

Soil prokaryotic community (SPC) composition was compared across samples using Bray-Curtis dissimilarity matrices generated from square root transformed rarefied ASV tables via non-parametric (distribution-free) permutational multivariate analysis of variance (PERMANOVA; “adonis2” function of the *vegan* package), with land use, drought scenarios and supplemented solution as factors and including sampling site as a covariate (Oksanen, 2019). All PERMANOVA analyses were conducted using sequential sum of squares under a reduced model with 999 permutations. Significant p-values in PERMANOVA shows a significant difference in either the centroids or the dispersion of the points in the multivariate space. Multivariate homogeneity for PERMANOVA factors was evaluated using the betadisper and permutest functions of the *vegan* package. To detect differences in SPC composition due to land use or drought scenarios the pairwise.adonis function of the *pairwiseAdonis* package was used, based on Bray-Curtis dissimilarity and using Bonferroni as a correction (Martinez Arbizu, 2020).

To visualize the beta-diversity patterns, we performed a nonmetric multidimensional scaling (NMDS) ordination using the metaMDS function (*vegan* package) based on a Bray-Curtis dissimilarity matrix. The ordination was constructed using species abundance data alone of the initial soils. To provide an exploratory visual context for the observed initial community patterns, environmental variables were passively fitted onto the final NMDS ordination plot using the envfit function (*vegan* package). Given the limited initial sample size (n=6), the fitting was used for qualitative illustration only to show the conditions associated with the predefined habitat types (forest and pasture) and not for formal statistical inference. The direction of each vector indicates the gradient of increasing value for that variable.

A linear discriminant analysis (LDA) effect size (LEfSe) approach was used to identify prokaryotic taxa that were significantly differentially abundant between each treatment group using the *microbiomeMarker* package. Only taxa with a > 3 log 10 LDA score were considered significantly enriched at a p-value < 0.05 (Kruskal-Wallis test). The Venn diagram was calculated with *eulerr* v.7.0.2 (Larsson and Gustafsson, 2018), *limma* v.3.58.1 (Ritchie et al., 2015), and *VennDiagram* v.1.7.3 (Chen, 2022).

Graphical displays of NMDS, relative abundance, and boxplot diagrams were generated using the *vegan* package in combination with *ggplot2* v.3.5.0 (Wickham, 2016) from the *tidyverse* collection.

## 3 Results

### 3.1 Initial composition of the prokaryotic community of old-growth forest and active pasture soils

**Figure 2:**
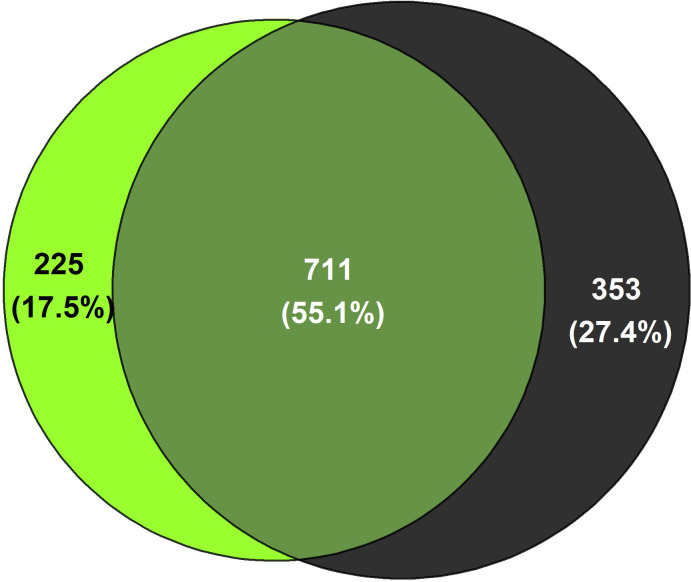
Number of prokaryotic ASVs unique to or shared between (intersections of cycles) forest (light green) and pasture soils (black) of the initial SPC.

Most of the measured parameters related to the physicochemical characteristics and nutrient status were similar in both ecosystems (**Supplementary Table S4**). However, the actual soil water content was significantly higher in forest soils (p=0.013) and δ^13^C (‰) was significantly more positive in pasture soils (p=0.040) dominated by C4 plants. The initial alpha diversity (Shannon index) and beta diversity (based on Bray-Curtis distances) did not differ between the SPC of forest and pasture soils (**Supplementary Table S4**; **Supplementary Figure S1A**). For the prokaryotic community, 55% of the 1289 ASVs identified in the initial SPC were shared between forest and pasture soils, and 225 and 353 ASVs were unique to forest and pasture soils, respectively (**Figure 2**). The most abundant phyla in the initial SPC of both ecosystems were Proteobacteria (FO: 26.2% relative abundance, PA: 21.9%), Acidobacteriota (FO: 23.2%, PA: 16.3%), and Chloroflexi (FO: 16.1%, PA: 17.8%) (**Supplementary Figure S1B**). Within the Proteobacteria phylum, most of the SPC was part of the Rhizobiales order (FO: 18.1%; PA: 16.3%). To estimate more detailed group-specific phyla, LEfSe analyses unveiled the enriched phyla with statistically significant differences (p < 0.05) in relative abundance between forest and pasture soils (**Supplementary Figure S2A**; **Supplementary Table S5**). Initial pasture soils had a differentially higher relative abundance of phyla Firmicutes (13.3%) and Actinobacteriota (15.4%) than forest soils (1.3% and 8.5%, respectively). In contrast, Crenarcheota (6.5%) and Nitrospirota (1.2%) were the phyla with the highest differential abundance in natural forest soils, compared to pasture systems (0.62% and 0.17%, respectively) (**Supplementary Figures S1B**, **S2B**; **Supplementary Table S5**).

At family level, the most abundant families in the initial SPC of both ecosystems were Xanthobacteraceae and Ktedonobacteracea followed by Acidothermaceae (**Supplementary Figure S3**). LEfSe analyses showed that at family level, the 4 most differentially abundant families (p < 0.05) between the SPC of pastures and forests were: Ktedonobacteraceae and Planococcaceae with a higher abundance in pasture soils, and Nitrososphaeraceae and Bathyarchaeia were more abundant in forest soils (**Supplementary Figure S2**; **Supplementary Table S6**). Other families that were differentially abundant between pasture and forest SPC were Bacillaceae, Acidothermaceae, Solirubrobacteraceae, and Streptomycetaceae, with the highest differential abundance in pasture systems, and Hyphomicrobiaceae, Solibacteraceae, and Nitrospiraceae, with a differentially higher abundance in forest soil (**Supplementary Figure S2**).

**Figure 3:**
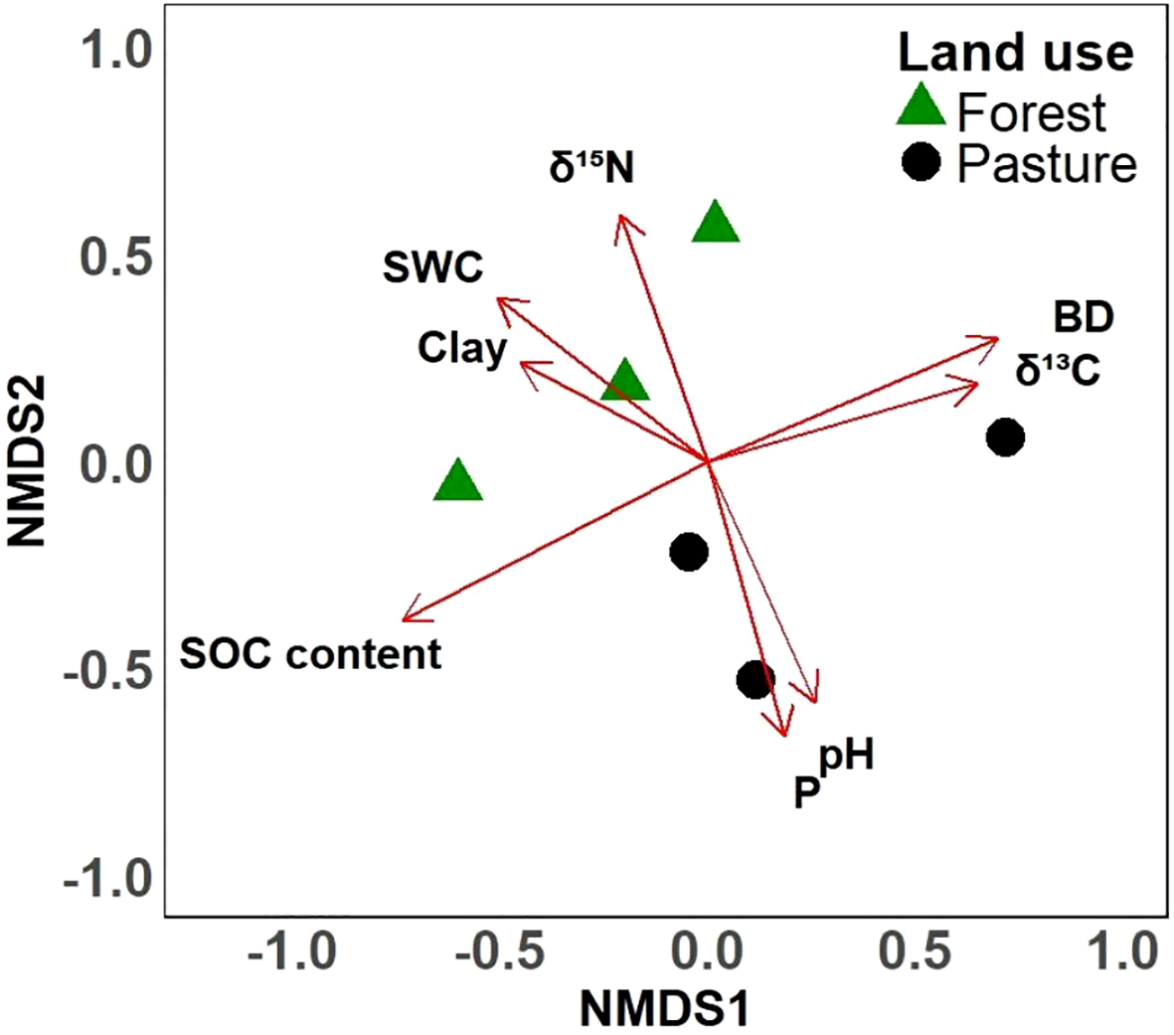
Non-metric multidimensional scaling (NMDS) ordination of prokaryotic community composition based on Bray-Curtis dissimilarity of initial soils before the incubation. The ordination (stress < 0.01) visualizes the dissimilarity between samples based on community data. Land use is distinguished by symbol color and shape: forests (green triangles) and pastures (black circles). To provide interpretive context, soil parameter were *post-hoc* fitted as vectors onto the ordination; the direction of each red arrow indicates the gradient of increasing values for that parameter. SOC content: soil organic carbon content (%), δ^15^N (‰), δ^13^C (‰), BD: bulk density (g cm^-3^), P: Plant available phosphorus (mg kg^-1^), Clay (%), SWC: soil water content (%).

Analysis on beta diversity data integrating environmental parameters in an exploratory NMDS (stress < 0.01) showed separation in multivariate space between the SPC of the forest and pasture sampling sites (**Figure 3**). For exploratory context, environmental variables were fitted to the ordination. Visually the vectors for soil water content, clay content, plant-available phosphorus and pH appeared to be associated with the observed compositional differences in the initial SPC structure between forest and pasture sites. However, due to the limited sample size (n=6), these correlations were not statistically significant, and thus these findings are exploratory and warrant cautions interpretation.

### 3.2 Activity of the soil prokaryotic community of pasture and old-growth forest soils during the incubation experiment

**Figure 4:**
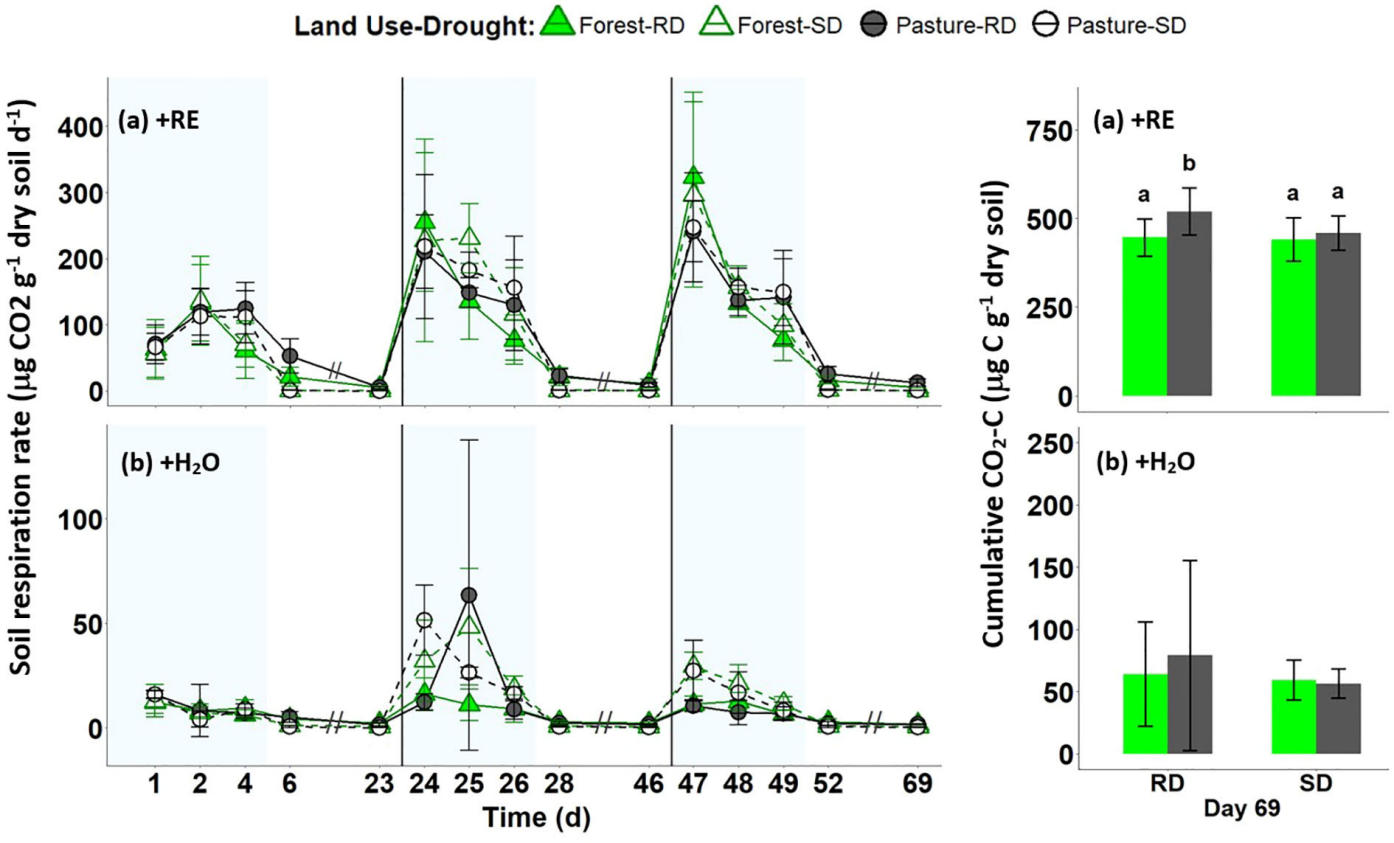
Dynamics of emission rates of carbon dioxide (CO_2_). **(A)** Upper graphs show the emission rates of the +RE treatment (artificial root exudate addition) and **(B)** lower graphs show the emission rates of +H_2_O treatment (no root exudate addition) in forest (green triangles) and pasture (black circles) soils during the whole incubation period. RD, reference drought (filled symbol - solid line) and SD, severe drought (empty symbol - dashed line). The three blocks demark the 3 drying-rewetting-cycles with reference in the background to the wet (light blue) and the dry cycle (white). The left graphs do not display the in-between days of the dry cycles since measurements were conducted only on the first and last day of the dry period. The bar graphs on the right represent the cumulative respiration on day 69 (mean ± standard deviation): in +RE different letters mean significant differences based on ANOVA and *post-hoc* Tukey’s test (F=6.04), p < 0.01; **(B)** The +H_2_O treatment showed no significant difference in the final cumulative respiration.

Rewetting of forest and pasture soils with artificial RE solution led to significantly higher CO_2_ emission rates during the wet period of each cycle along with the largest fluctuations in CO_2_ emissions between wet and dry periods compared to +H_2_O treatments (**Figure 4**; **Supplementary Table S7**). After the first drought event, CO_2_ evolution from forest soils increased and remained high in the following two wet periods in all the treatments, except for RD+H_2_O. In the pasture soils the same pattern was detected for the +RE treatments, but in the +H_2_O treatments after the second cycle CO_2_ emissions decreased again in the third wet period. During the dry period, both soils subjected to +RE showed significantly lower CO_2_ emissions in the SD treatment (5% WHC) than those soils that were subjected to RD of 30% WHC (**Figure 4**; **Supplementary Table S7**). At the end of the experiment the soils subjected to +RE emitted around 4.22% of the initial C (endogenous + supplemented C), while the soils subjected to +H_2_O emitted around 0.74% of the initial endogenous C (**Supplementary Table S8**), which represents a 7.2-fold increase in cumulative soil respiration in the +RE treatments compared to the +H_2_O treatments.

### 3.3 Diversity and structure of soil prokaryotic community of pasture and old-growth forest soil under drought stress and artificial root exudate addition

A total of 1559 ASVs with a relative abundance of more than 0.003% were obtained from the undisturbed soils samples of three active pastures and three old-growth forests, as well as 81 soil samples after the incubation experiment. From this dataset, we calculated the following alpha diversity indices: Shannon, Simpson, Chao1 indices, observed ASVs and evenness index (**Table 1**; **Supplementary Table S9**). Although the initial alpha diversity did not differ between forest and pasture soils (see section 3.1), the temporal changes in alpha-diversity were significant after SD events. The SPC of the SD+RE treatment showed the lowest diversity values, except for Simpson’s index, where values were also significantly lower for the SD+H_2_O incubation units of forest soils (**Supplementary Table S9**). A comparison of Bray-Curtis showed that forest soils under the SD+RE treatment diverged more from the initial SPC than the other treatments (**Supplementary Figure S4**). In the pasture soils, both RD+RE and SD+RE caused significant shifts in the SPC composition compared to the initial stage (**Supplementary Figure S4**).

**Table 1:** Mean α-diversity indices (non-parametric Shannon diversity index; Chao1 richness estimator and Evenness index) at different treatments following the incubation experiment (mean ± standard deviation).

Treatment	n	Shannon index	Chao index	Evenness index
Initial soils				
Forest	3	5.5 ± 0.2ab	535 ± 83abc	0.877 ± 0.01a
Pasture	3	5.6 ± 0.1ab	601.5 ± 81ab	0.871 ± 0.01a
Reference drought				
Forest +H_2_O	6	5.1 ± 0.2a	561 ± 35ab	0.815 ± 0.03a
Pasture +H_2_O	6	5.4 ± 0.3a	650 ± 49a	0.835 ± 0.05a
Forest +RE	15	4.0 ± 0.4abc	514 ± 41b	0.645 ± 0.06ab
Pasture +RE	14	4.0 ± 0.3abc	457 ± 87bcd	0.664 ± 0.05ab
Severe drought				
Forest +H_2_O	6	3.5 ± 0.5bcd	451 ± 79bcd	0.579 ± 0.07b
Pasture +H_2_O	6	4.0 ± 0.5abcd	496 ± 113bc	0.656 ± 0.07ab
Forest +RE	14	3.3 ± 0.5d	364 ± 73 d	0.558 ± 0.08b
Pasture +RE	14	3.5 ± 0.3cd	390 ± 110cd	0.599 ± 0.03b
		X^2^=61.676	F=10.44	X^2^=56.42

+H_2_O, only water was added. +RE, root exudate solution. Different letters in the same column mean significant difference based on ANOVA and *post-hoc* Tukey’s test (F) or Kruskal-Wallis and *post-hoc* Dunn test (X^2^); p-value < 0.001.

**Figure 5:**
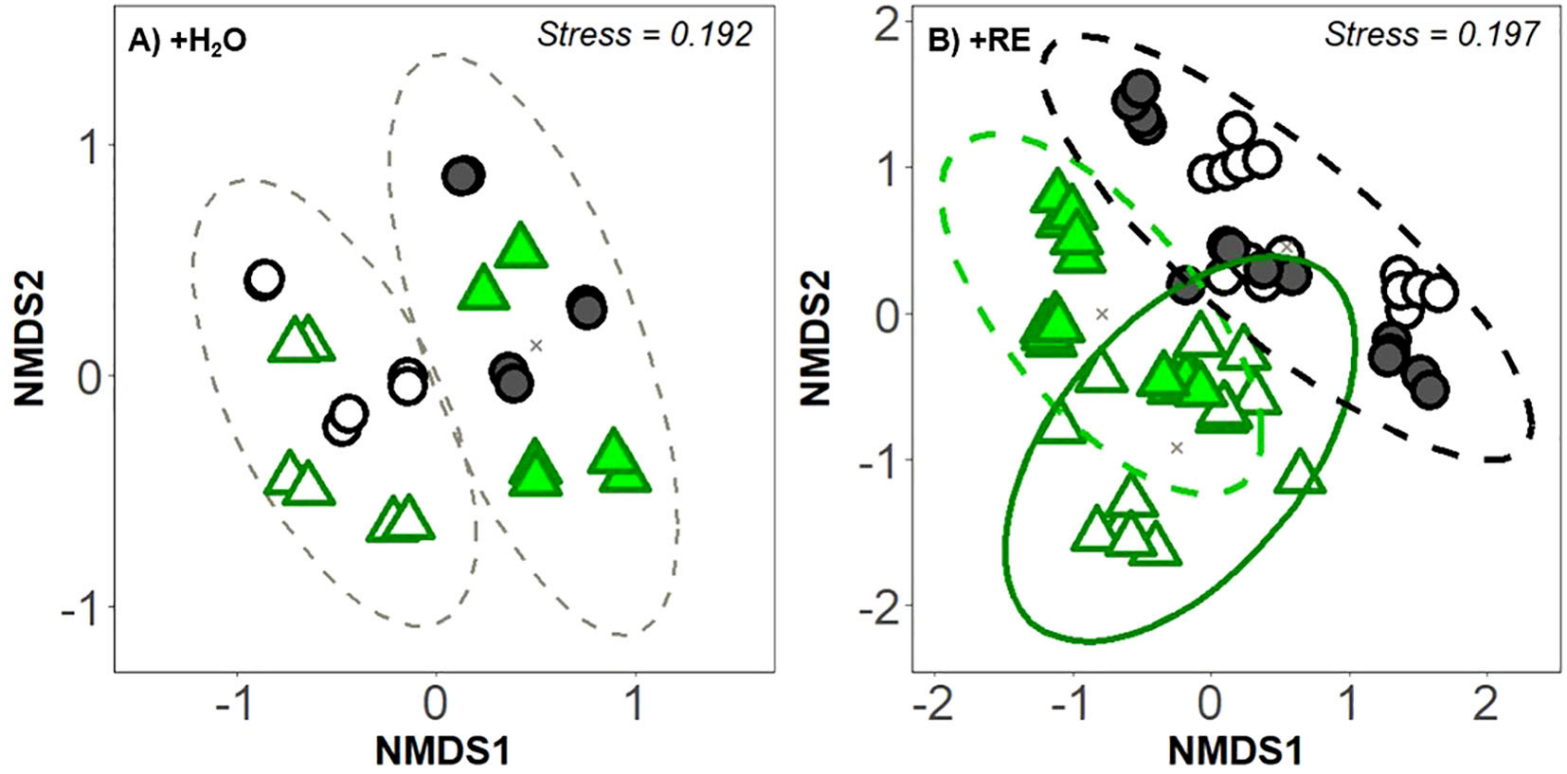
Non-metric multidimensional scaling (NMDS) ordination of prokaryotic community composition based on Bray-Curtis dissimilarity for the **(A)** +H_2_O treatment, without RE application and **(B)** +RE treatments. Land use is distinguished by symbol color and shape: forest (green triangle) and pasture (black circle). Symbol filling represents drought scenarios, RD, reference drought (filled), SD, severe drought (empty). Ellipses represent the 95% confidence intervals for group clusters. Significant group differences (p < 0.05) were confirmed by PERMANOVA (**Supplementary Table S10**).

A factorial PERMANOVA of the pooled data including the temporal data (initial and final SPC) and +RE treatment as factors was not possible due to unequal sample sizes and heterogeneous dispersion between groups (permutest: p < 0.05). Two separate PERMANOVA analyses of +H_2_O and +RE treatments revealed for both the composition of SPCs at the end of the experiment was significantly influenced by drought intensity and land use (**Figure 5**; **Table 2**; PERMANOVA: p < 0.001). Community composition also varied significantly across the sampling sites (**Table 2**; PERMANOVA: p < 0.001).

**Table 2:** PERMANOVA analysis of prokaryotic community composition in incubation units (with or without root exudate addition) after the experiment (999 permutations).

PERMANOVA					
Water (+H_2_0)					
Factors	df	SS	Pseudo-F	P	% Variance explained
Drought intensity	1	1.66	32.61	***	27.37
Drought intensity X Land-use type	1	0.10	1.91	0.077	1.61
Land-use type	1	0.86	16.94	***	14.22
Site (covariate)	4	2.63	12.92	***	43.38
Residuals	16	0.82			13.43
Total	23	6.08			100
RE addition (+RE)					
Factors	df	SS	Pseudo-F	P	% Variance explained
Drought intensity	1	1.64	23.31	***	10.65
Drought intensity X Land-use type	1	0.54	7.72	***	4.71
Land-use type	1	2.81	40.01	***	18.28
Site (covariate)	4	6.95	24.71	***	45.16
Residuals	49	3.45			22.39
Total	56	15.39			100

p < 0.001 as ***. RE, root exudates.

In the +H_2_O treatment, the influence of drought intensity was greater (27.4% of variance explained) than that of land use (14.2% of variance explained) in shaping the prokaryotic community at the end of the experiment (**Table 2**; **Figure 5A**). Additionally, pairwise PERMANOVA indicated a significant difference in the SPC composition between drought intensity treatments, but not between land use (**Supplementary Table S10**). On the contrary, in the soil samples supplemented with artificial RE solution the influence of drought intensity (10.7% of variance explained) was smaller than that of land use legacy (18.3% of variance explained) in shaping the prokaryotic community. At RE addition, the interaction between drought intensity and land use significantly influenced (4.7% of variance explained) the final SPC composition (**Table 2**, **Figure 5B**). In this case, pairwise PERMANOVA indicated a significant difference in SPC composition between land-use type (**Supplementary Table S10**; **Figure 5B**). In addition, the forest soils also showed a significant difference in the SPC composition between drought intensity treatments.

### 3.4 Response pattern of soil prokaryotes under induced drought stress and artificial root exudate addition

**Figure 6:**
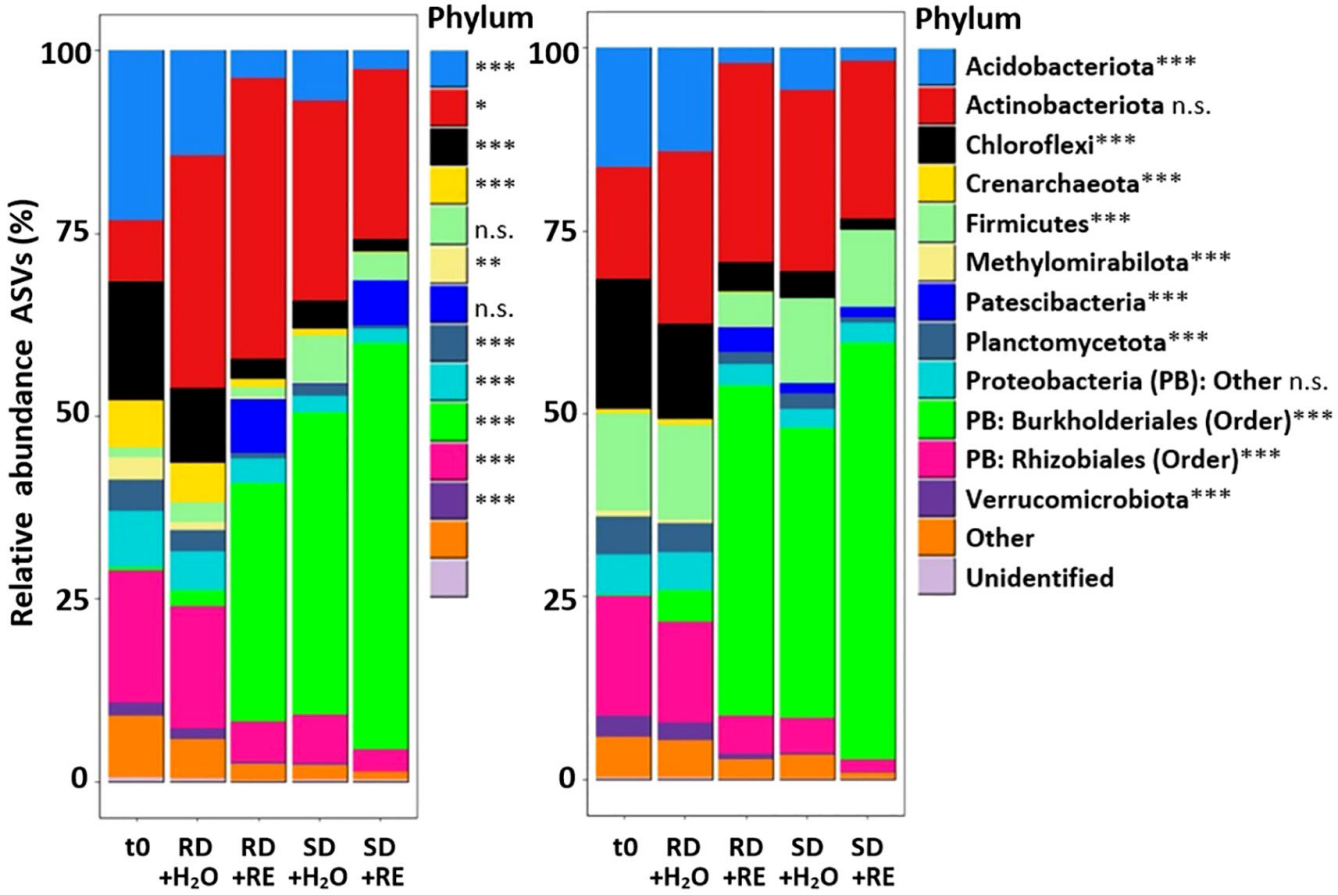
Relative abundance of 10 most abundant prokaryotic taxa (phylum) under different drought and root exudate (RE) addition treatments of forest (left) and pasture (right) soils. t0: initial community; RD+H_2_O (mean reference drought – no root exudate addition); RD+RE (mean reference drought + root exudate addition); SD+H_2_O (severe drought – no root exudate addition); SD+RE (severe drought + root exudate addition). “Other” taxon includes 13 phyla with a relative abundance below 1.5% in both ecosystems: Armatimonadota, Bacteroidota, Bdellovibrionota, Cyanobacteria, Desulfobacterota, GAL 5, Gemmatimonadota, Halobacterota, Myxococcota, NB-j, Nitrospirota, RCP2–54 and WPS-2. Asterisks indicate statistically differences in forest or pasture soils after the incubation: *p < 0.05, **p < 0.01, ***p < 0.001, n.s. (not significant).

Drought stress and artificial RE addition significantly affected (p < 0.01) the overall relative abundance of the following phyla in both ecosystems: Acidobacteriota, Chloroflexi, Crenarcheota, Methylomirabilota, Planctomycetota, and Verrucimicrobiota, and the two most abundant orders within the phylum Proteobacteria: Burkholderiales and Rhizobiales (**Figure 6**, **Supplementary Table S5**). Moreover, in forest soils, the relative abundance of Actinobacteriota (p < 0.05) and the combination of the less abundant orders of Proteobacteria phylum (p < 0.001) were also affected by drought and artificial RE addition. In pasture soils, the relative abundance of Firmicutes and Patescibacteria differed significantly between the treatments (p < 0.001). The soil prokaryotic communities showed no significant differences before and after the incubation for the RD+H_2_O treatment at phylum or family level in either of the two tropical ecosystems studied (**Supplementary Table S5**).

In forest soil, the relative abundance of some prokaryotic phyla was significantly decreased after RD+RE, SD+H_2_O, and SD+RE treatments in comparison to the initial SPC (t0): Acidobacteriota, Crenarcheota, Planctomycetota, Verrucomicrobiota, and the order Rhizobiales (**Figure 6**; **Supplementary Table S5**). The relative abundance of Chloroflexi significantly decreased with the addition of RE, after both RD and SD. The relative abundance of Methylomirabilota was significantly decreased after SD+RE treatment. In contrast, Burkhoderiales (order within the Proteobacteria phylum) were positively affected by SD accounting for around 40-55% of the relative abundance in the SD+H_2_O and SD+RE treatments in forest soil. Moreover, the relative abundance of Actinobacteriota was significantly higher in the RD+RE treatment than in the initial SPC sample.

In pasture soils, the relative abundance of Burkhoderiales (order within the Proteobacteria phylum) was significantly increased by artificial RE addition accounting for around 45-55% of the relative abundance in the RD+RE and SD+RE treatments (**Figure 6**; **Supplementary Table S5**). Patescibacteria were positively affected by RD+RE, SD+H_2_O, and SD+RE treatments. Conversely, the relative abundance of some prokaryotic phyla was significantly decreased after RD+RE, SD+H_2_O, and SD+RE treatments in comparison to the initial SPC of pastures, which was the case for the phyla Chloroflexi, Methylomirabilota, Verrucomicrobiota, and the order Rhizobiales. The relative abundance of Acidobacteriota significantly decreased with the addition of REs after both reference and severe drought. The relative abundance of Crenarcheota and Planctomycetota significantly decreased in the SD+RE treatment, whereas the relative abundance of Firmicutes was significantly lower in the RD+RE treatment than in the initial SPC of the pastures.

At family level, the most abundant families changed from Xanthobacteraceae and Ktedonobacteraceae in the initial SPC (t0) to Oxalobacteraceae and Micrococcaceae at the end of the experiment (**Supplementary Figure S3**). In both ecosystems, the relative abundance of Burkholderiaceae significantly increased after RD+RE, and Oxalobacteraceae significantly increased after severe droughts (SD+H_2_O and SD+RE), compared to the initial SPC (**Supplementary Figure S3**; **Supplementary Table S6**). Xanthobacteraceae significantly decreased in pastures after RD+RE, SD+H_2_O, and SD+RE treatments. In forests, Xanthobacteraceae significantly decreased only after SD+RE treatment, whereas Ktedonobacteraceae significantly decreased after SD+RE treatment in both ecosystems.

## 4 Discussion

### 4.1 Differences in initial prokaryotic communities between old-growth forests and active pasture soils

The initial SPCs of old-growth forests and pastures exhibited distinct compositional profiles, shaped by decades of contrasting land-use practices. Pasture soils were enriched in phyla associated with drought resistance, such as Firmicutes (13.3% vs. 1.3% in forests) and Actinobacteriota (15.4% vs. 8.5%), which are known for their physiological adaptations to water stress, including spore formation and thick cell walls (Naylor and Coleman-Derr, 2018; Paes da Costa et al., 2022). These taxa thrive in the harsher microclimates of pastures, where the absence of canopy cover, soil compaction from cattle trampling, and lower soil moisture (11.1% vs 23.1% in forests) create selective pressure for drought-tolerant lineages (Nepstad et al., 1996; Souza et al., 2016). In contrast, forest soils harbored a higher abundance of Crenarcheaota (6.5% vs. 0.6%) and Nitrospirota (1.2% vs. 0.2%), taxa critical for nitrogen cycling, including ammonia-oxidizing archaea (Nitrososphaeraceae) and nitrite-oxidizing bacteria (Nitrospiraceae) (Daims et al., 2016; Pedrinho et al., 2019). The prevalence of these nitrifiers aligns with the more stable, organic-rich conditions of forest soils, which support complex nutrient cycling networks (Davidson et al., 2000; Vitousek, 1984).

Land-use legacies further influenced soil physicochemical properties. Pasture soils tend to have lower clay content (24.2% vs. 33.2%), likely due to erosion (Martínez and Zinck, 2004; Veldkamp et al., 2020), slightly higher pH (4.5 vs. 4.3), and higher plant-available P (7.9 vs 2.5 mg kg^-1^). Although soil pH can influence SPC composition by affecting nutrient availability and physiological stress (Lammel et al., 2018; Tripathi et al., 2012), its role as a distinguishing factor was likely limited in our study, as differences between land uses were small and not statistically significant (**Supplementary Table S4**). The efficient uptake and recycling of P by plants and microorganisms in forests may contribute to the lower levels of plant-available P in the forest soils (Vitousek and Sanford, 1986).

The relationship between environmental variables and SPC composition operated differently between land uses. In pastures, the combination of low soil moisture and coarser texture might have created strong selective pressure for drought-resistant taxa. Conversely, forest communities responded to the more stable, fine-textured, moisture-rich and carbon-rich conditions by supporting specialized nutrient-cycling assemblages. The different environmental conditions resulted in distinct ecological filtering patterns that shaped community composition in each land use type. These differences underscore how long-term deforestation alters abiotic conditions and microbial ecology, priming pasture SPCs for resilience to future droughts, a critical precursor to testing hypothesis 1.

### 4.2 Response of soil prokaryotic communities to drought without root exudate addition

Both forest and pasture SPCs showed resistance to reference drought (RD+H_2_O treatment) of southwestern Amazonia, with no significant effect on diversity and composition (**Table 1**; **Figure 6**). The evenness of SPCs remained high, indicating stability in both ecosystems. Soils were sampled at the end of the dry season when water content at 15 cm depth in both ecosystems was lower than that of the RD+H_2_O treatment during dry periods. Thus, SPCs of both land uses were already well-adapted to regional dry season (Zhou et al., 2016), with matric potential below -15.000 hPa.

In contrast, severe drought stress (SD+H_2_O treatment) modified SPC structure differently between land uses, supporting hypothesis 1. Forest soils experienced greater compositional alterations than pasture (**Supplementary Table S5**), with evenness in forest soils decreasing from 0.87 to 0.58 (**Table 1**), highlighting the pressure on certain forest species to acclimatize. Diversity experienced minimal decline in both land uses. This observation aligns with previous studies reporting minor effects of drought on microbial diversity and richness, but profound shifts in SPC composition (Barnard et al., 2013; Naylor and Coleman-Derr, 2018). The resilience observed in prokaryotic diversity was presumably due to species unable to acclimatize continuing to be present in low numbers, with only a few occurring below detection limit (Baas-Becking, 1934; Schimel et al., 2007).

Cumulative soil respiration was similar between land uses after both drought treatments (**Figure 4**), consistent with previous studies (Neill et al., 1996; Schwendenmann and Pendall, 2008), though slightly lower possibly due to our soil sampling from a deeper soil layer. The Birch effect, a CO_2_ pulse following rewetting, was evident across cycles in both systems, driven by the rapid reactivation and activity of surviving taxa (Birch, 1958; Schimel, 2018). CO_2_ emission rates remain stable between cycles after each dry period, indicating SPC adaptation to drought stress when no additional carbon source is provided to microorganisms in both land uses.

Regarding SPC adaptation, both land uses developed similar dominance of Oxalobacteraceae (order Burkholderiales, phylum Proteobacteria) after SD+H_2_O treatment, accounting for over one-third of total relative abundance. Many Oxalobacteraceae members are not only drought-tolerant bacteria (Maestro-Gaitán et al., 2023; Monohon et al., 2021; Ofek et al., 2012), but also promote plant growth under drought (Lei et al., 2023; Mickan et al., 2019). Firmicutes and Actinobacteriota – initially more abundant in pasture than forest soils – proved resistant to water deficit (Canarini et al., 2024; Naylor and Coleman-Derr, 2018), maintaining abundance similar to initial levels. Nonetheless, contrary to our findings, most studies showed significant increase in relative abundance of these gram-positive phyla post-drought stress (Dacal et al., 2022; Dai et al., 2019; Jaeger et al., 2024; Naylor and Coleman-Derr, 2018). Our samples might have exhibited initially increased abundance of drought-resistant microorganisms due to collection during the dry season.

Conversely, drought intensification significantly reduced sensitive taxa such as Verrucomicrobiota (Dai et al., 2019; Moore et al., 2023; Siebielec et al., 2020), and order Rhizobiales in both ecosystems, while Chloroflexi decreased (Dai et al., 2019) specifically in pasture soils. The fact that these groups declined from a baseline of already high seasonal stress indicates that drought intensification can push tropical soil communities beyond their seasonal adaptative capacity, leading to loss of taxonomic and likely functional diversity that is not recovered simply by the pre-existing presence of resistant taxa. This dominance of few phyla, likely reduces functional redundancy, negatively affecting ecosystem processes (Baldrian et al., 2023; Bérard et al., 2015).

Under +H_2_O treatments, drought intensity influenced SPCs more than land use (**Table 2**), yet response magnitude varied between ecosystems. This differential response reflects contrasting environmental-community relationships. In pastures, SPC seemed less impacted by severe drought, possibly owing to historical exposure to drier conditions, which fostered selection of drought-adapted taxa (Bouskill et al., 2013; Canarini et al., 2021; Moreno et al., 2019). Forest soils, which naturally maintain higher water content (Nepstad et al., 1996; Pedrinho et al., 2019; Souza et al., 2016; Vitousek, 1984), due to deeper roots (Nepstad et al., 1996; Vitousek, 1984), thicker organic layers, and canopy protection against evaporation (Nepstad et al., 1996; Pierret et al., 2016; Rawls et al., 2003), seemed to experience more pronounced shifts when drought exceeded their adaptive thresholds. This suggests that land-use legacy creates differential vulnerability patterns, with forests showing greater sensitivity to drought intensification despite their natural water management characteristics.

### 4.3 Root exudate addition modulates drought impacts on soil prokaryotic communities

Artificial root exudate addition amplified microbial activity and intensified drought-driven community shifts, corroborating hypothesis 2. The +RE treatments increased cumulative CO_2_ emissions by 7.2-fold compared to +H_2_O across both ecosystems (**Supplementary Table S7**), with emissions peaking during wet phases as microbes rapidly metabolized labile carbon (Naylor and Coleman-Derr, 2018; Schimel et al., 2007). This resource pulse favored opportunistic taxa like Actinobacteriota and order Burkhoderiales, the latter increasing by 40-55% in relative abundance under severe drought (**Figure 6**). These taxa are adept at exploiting simple carbohydrates and amino acids (Hervé et al., 2016; Lopes et al., 2022), traits that likely conferred a competitive advantage under fluctuating moisture.

Root exudate addition modestly enhanced Oxalobacteraceae growth under reference drought conditions, increasing from near-zero initial abundance to 7.5% in forests and 15.3% in pastures compared to 1.5% and 4.0% in RD+H_2_O treatments respectively (**Supplementary Table S6**). However, severe drought stress, rather than RE availability per se, drove the major community restructuring towards Oxalobacteraceae dominance (38-45% dominance in both SD+H2O and SD+RE treatments across both systems). The dominance of Oxalobacteraceae under severe drought reflects their physiological adaptation to water stress (Lei et al., 2023; Monohon et al., 2021), being even detected in high abundance in deserts (Nagy et al., 2005). Some members are plant growth-promoting bacteria that solubilize inorganic P (Baldani et al., 2014; Estrada et al., 2013), and promote nitrogen acquisition by plants during N deficiency (Baldani et al., 2014; Yu et al., 2021), important traits in nutrient-limited tropical soils. Burkholderiaceae increased significantly under RD+RE conditions, reaching 25.2% in forests and 29.9% in pastures compared to minimal abundance after +H_2_O treatments. Their higher abundance after RD+RE than SD+RE treatments suggests some members are well-adapted to exploit episodic resource pulses but are susceptible to drought intensification. Hervé et al. (2016) reported oxalotrophy within Burkholderiaceae, thus bacteria could be utilizing not only the supplemented trehalose and leucine, but also the oxalic acid.

The SD+RE treatments accelerated the decline of initially dominant families. Xanthobacteraceae decreased from 14.4% to 2.6% in forests and from 14.6% to 1.3% in pastures, while Ktedonobacteraceae declined from 5.9% to 0.5% in forests and from 10.2% to 0.8% in pastures. Chloroflexi and Planctomycetota - phyla associated with organic matter decomposition - declined by >50% in +RE treatments in both ecosystems (**Figure 6**, **Supplementary Table S5**), potentially impairing carbon cycling under prolonged drought. Rhizobiales (key nitrogen fixers) declined by 60% under SD+RE. These patterns suggest RE addition intensified competitive exclusion of slower-growing taxa under drought stress in both land uses. Under the combined stress of drought and resource pulses, these taxa likely cannot compete with rapid growth and resource acquisition strategies of copiotrophic species within Oxalobacteraceae and Burkholderiaceae (Fierer et al., 2007; Naylor et al., 2022). The result are homogenized communities dominated by a few highly competitive, stress-tolerant taxa in both land uses. Notably, Firmicutes was the only phylum that showed opposite directional responses between land uses: its abundance tended to increase from low initial levels in forests (1.33% to a maximum of 6.4%, non-significant) but decrease in pastures from high initial levels (13.3% to 4.61% under RD+RE, p < 0.001). This pattern likely reflects the relationship between initial abundance and competitive dynamics. In old-growth forests, rare drought-tolerant Firmicutes species may have opportunistically expanded into unoccupied niches. Conversely, their higher initial abundance in pastures likely made them more vulnerable to displacement by RE-stimulated copiotrophs.

RE addition intensified community homogenization, reducing Shannon diversity by 30-40% in severe drought treatments (**Table 1**), supporting hypothesis 2’s prediction of a trade-off between stress tolerance and diversity. While our experimental setup does not replicate a natural long-term drought gradient, it provides mechanistic evidence that this trade-off can be driven by the interaction of water stress and labile carbon availability in both land uses. REs stimulated activity while increasing selection for a narrow cohort of drought-adapted taxa, likely eroding functional redundancy. Our results support that, under natural conditions, not only soil water content but also other ecological factors (e.g.: nutrient availability and RE composition) would likely play important roles in shaping SPC dynamics under drought stress. Root exudate inputs may disrupt mutualistic networks in historically stable ecosystems, with cascading effects on nutrient availability (Qiu et al., 2023). However, it is important to consider the composition of our artificial RE solution. While our simplified mixture of trehalose, leucine, and oxalic acid is based on common RE components, it cannot represent the full chemical diversity of the REs from complex tropical forest ecosystems, and the specific artificial RE composition might have favored species adapted to utilize these particular compounds. Future research could investigate how RE profiles of specific tropical tree species modulate this interaction.

Land-use legacy modulated RE effects differently between ecosystems. Pasture SPCs exhibited smaller compositional shifts than forests SPCs after drought intensification (**Figure 5B**, **Supplementary Table S10**), likely due to pre-existing dominance of drought-tolerant taxa (e.g. Firmicutes). Under +RE treatments, PERMANOVA analysis revealed that land legacy (18.3% of variance explained) had greater influence than drought intensity (10.7%) on SPC composition, contrasting with +H_2_O treatments where drought intensity dominated (27.4% vs. 14.2%). This suggests that carbon availability interacts with land-use specific environmental properties and partially buffers drought effects, particularly in pasture soils. Consequently, faster recovery rates after drought stress are expected in the pasture SPC (Barnard et al., 2013; Bouskill et al., 2013), particularly under high carbon and nutrient availability conditions.

### 4.4 Synthesis: implications for ecosystem resilience

To ensure a comprehensive understanding of the implications of the study for ecosystem resilience, we acknowledge certain methodological considerations. Microcosms controlled confounding variables; however, the absence of plant roots and natural exudate dynamics limits ecological extrapolation. For instance, live roots modulate exudate composition in response to stress (Maurer et al., 2021; Vives-Peris et al., 2020), a feedback absent in our experiment. Additionally, field experiments are advised to validate trends observed in our microcosms and further experiments using DNA stable isotope probing (DNA-SIP) or multi-omics (e.g., metatranscriptomics) could clarify which microorganisms assimilate the provided artificial REs and their functions under drought stress. While considering these methodological aspects, our findings validate both hypotheses but highlight complex interactions between drought intensity, land use, and carbon inputs. Hypothesis 1 is partially supported, as severe drought reduced forest SPC diversity and altered composition more than in pastures. Hypothesis 2 holds true, REs amplified drought-driven community shifts, but land-use legacy buffered these effects in pasture, underscoring the importance of land use history in microbial responses.

The dominance of a few drought-resistant taxa post-treatment raises critical implications in ecosystem stability and climate feedbacks. The decline in taxa central to soil carbon and nitrogen cycling (e.g., Acidobacteriota and Rhizobiales) may impair long-term nutrient retention (Dedysh and Damsté, 2018; Qiu et al., 2023). However, the rise of Burkholderiales could enhance short-term stress mitigation through plant growth promotion (Ofek et al., 2012). Regarding climate feedbacks, the observed increase in CO_2_ emissions from RE-amended soils (**Supplementary Table S6**) suggests that drought-stressed plants may inadvertently amplify soil carbon losses through exudate-driven microbial activation (Preece and Peñuelas, 2016). The projected changes in drought patterns poses increased stress risks for tropical soils. Our results reinforce the hypothesis that drought intensification may destabilize SPCs and hinder the optimal functioning of soil ecosystems, e.g. by altering nutrient cycling, pathogen control, and organic matter decomposition.

## 5 Conclusion

Our incubation experiment suggests that tropical soil prokaryotic communities are resistant to current drought regimes but vulnerable to drought intensification, with land use legacy shaping these responses. Pasture SPCs exhibit greater resistance to severe drought due to historical stress exposure and pre-adaptation through initial higher levels drought-tolerant taxa (Firmicutes, Actinobacteriota), while forest communities experience greater compositional disruption when drought exceeds their natural adaptive thresholds. The impact of drought intensification on SPCs extends beyond simple water limitation, triggering community restructuring towards dominance by a few stress-tolerant families, particularly Oxalobacteraceae. This taxonomic homogenization might reduce functional redundancy, with sensitive taxa critical for nitrogen cycling (Rhizobiales) declining significantly across both land uses. Root exudates emerge as a double-edge sword: they sustain microbial activity but increase community homogenization and intensify competitive exclusion of slower-growing oligotrophic species. Critically, land-use legacy modulates these effects, with carbon availability buffering drought impacts more effectively in pasture systems. Under RE addition, land use becomes a stronger predictor of community composition than drought intensity, underlining how legacy shapes microbial community. These results highlight the importance of considering the complex interactions between plants and microorganisms to better understand and predict ecosystem resilience under changing climate conditions.

## Data Availability

The datasets presented in this study can be found in online repositories. The names of the repository/repositories and accession number(s) can be found below: https://www.ncbi.nlm.nih.gov/, PRJNA1242199. The raw data, metadata and analysis scripts supporting this study are openly available on Zenodo at DOI: 10.5281/zenodo.15094711.
