# Supplementary material for: Adaptation and resistance of soil prokaryotic communities to drought intensification in old-growth forests and pastures of southwestern Amazonia
Source: Front Plant Sci. 2025 Nov 3;16:1684321. doi: 10.3389/fpls.2025.1684321 (PMC12621651; doi:10.3389/fpls.2025.1684321)
Supplement: Supplementary file 1 [file DataSheet1.docx]

**Supplementary Material**

| **Table S.1** **\|** Overview of the incubation treatments per land use. For both land uses, the incubation experiment includes 3 sampling sites as spatial repetitions, as well as a combination of 2 drought scenarios and 2 solutions for the rewetting of the soils. | | | |
| --- | --- | --- | --- |
| **Incubation treatments** | **Drought scenarios** | **Supplemented solution** | **Number of incubation units per sampling site** |
| RD + H_2_O | Reference: 30% WHC | Water | 2  5  2  5 |
| RD + RE | Reference: 30% WHC | Artificial root exudate |  |
| SD + H_2_O | Severe: 5% WHC | Water |  |
| SD + RE | Severe: 5% WHC | Artificial root exudate |  |

| **Table S.2 \|** Calculated matric potentials based on pedotransfer-functions at Tomasella & Hodnett (1998) and Van-Genuchten equation as a mean for each land use level. This approach effectively models unsaturated soils but loses reliability in extreme dryness, as observed at 5% WHC in this study, where matric potentials could not be calculated. | | | | | | |
| --- | --- | --- | --- | --- | --- | --- |
|  |  | **Drought steps (% WHC)** | | | | |
| **Land use** |  | **95** | **75** | **50** | **30** | **5** |
| **Old-growth forest** | theta [m^3^/m^3^] | 53.2 | 42 | 28 | 16.8 | 2.8 |
|  | pF | 1.55 | 2.29 | 3.41 | 5.22 | - |
|  | pF [hPa] | -35.5 | -195.0 | -2570.4 | -165958.7 | - |
| **Pasture** | theta [m^3^/m^3^] | 44.65 | 35.25 | 23.5 | 14.1 | 2.35 |
|  | pF | 1.2 | 1.92 | 2.87 | 4.63 | - |
|  | pF [hPa] | -15.8 | -83.2 | -741.3 | -42658.0 | - |

| **Table S.3** **\|** Van-Genuchten parameters for calculation of soil hydraulic properties based on α as the inverse of the air entry suction, n as the pore size distribution, θs as the saturated water content, θr as the residual water content, and m as the van Genuchten fitting parameter. | | | | | |
| --- | --- | --- | --- | --- | --- |
| **Land use** | **α** [cm^-1^] | **n** | **θs** [m³/m³] | **θr** [m³/m³] | **m** |
| **Old-growth Forest** | 0.03 | 1.23 | 60.07 | 9.92 | 0.19 |
| **Pasture** | 0.04 | 1.31 | 48.68 | 10.28 | 0.23 |

| **Table S.4 \|** Soil properties of the initial soils (mean ± standard deviation). Bolded text and asterisk indicate significant difference between old-growth forest and pasture soils based on Welch t-test (p < 0.05). δ^13^C (‰): t=3.0 and SWC (%): t=3.9. | | |
| --- | --- | --- |
| **Soil properties** | **Forest**  (n=3) | **Pasture**  (n=3) |
| pH (H_2_O) | 4.3 ± 0.2 | 4.5 ± 0.2 |
| Sand (%) | 18.4 ± 13.6 | 45.5 ± 19.2 |
| Silt (%) | 48.3 ± 12.0 | 30.3 ± 12.6 |
| Clay (%) | 33.2 ± 5.4 | 24.2 ± 8.2 |
| C content (mg g^-1^) | 9.6 ± 2.5 | 8.2 ± 1.9 |
| N content (mg g^-1^) | 1.3 ± 0.3 | 1.0 ± 0.4 |
| C:N ratio | 7.3 ± 2.1 | 8.8 ± 1.7 |
| δ^15^N (‰) | 11.5 ± 4.1 | 8.1 ± 2.3 |
| **δ^13^C (‰) *** | **-26.4 ± 1.4** | **-23.0 ± 1.4** |
| CEC | 5.2 ± 2.1 | 3.0 ± 1.1 |
| Plant available nutrients | |  |
| *Al (mg kg^-1^)* | 1275.1 ± 283.0 | 794.6 ± 252.6 |
| *Fe (mg kg^-1^)* | 246.1 ± 118.8 | 221.0 ± 27.7 |
| *P (mg kg^-1^)* | 2.5 ± 1.1 | 7.9 ± 8.2 |
| *Ca (mg kg^-1^)* | 35.6 ± 46.7 | 112.0 ± 111.4 |
| *Mg (mg kg^-1^)* | 61.3 ± 94.1 | 32.7 ± 25.3 |
| *K (mg kg^-1^)* | 32.6 ± 18.6 | 98.7 ± 80.1 |
| *Mn (mg kg^-1^)* | 169.0 ± 244.1 | 35.1 ± 40.1 |
| **SWC (%) *** | **23.1 ± 3.7** | **11.1 ± 3.2** |
| BD (g cm^-3^) | 1.1 ± 0.2 | 1.2 ± 0.1 |
| Shannon Index | 5.5 ± 0.2 | 5.6 ± 0.1 |


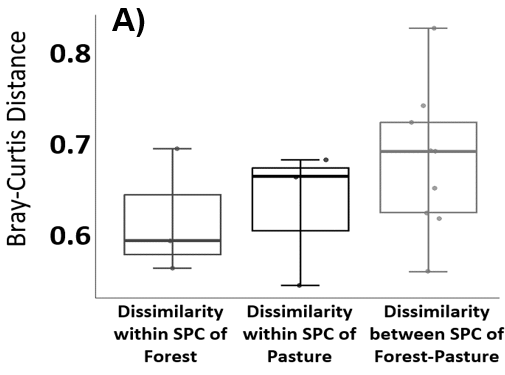

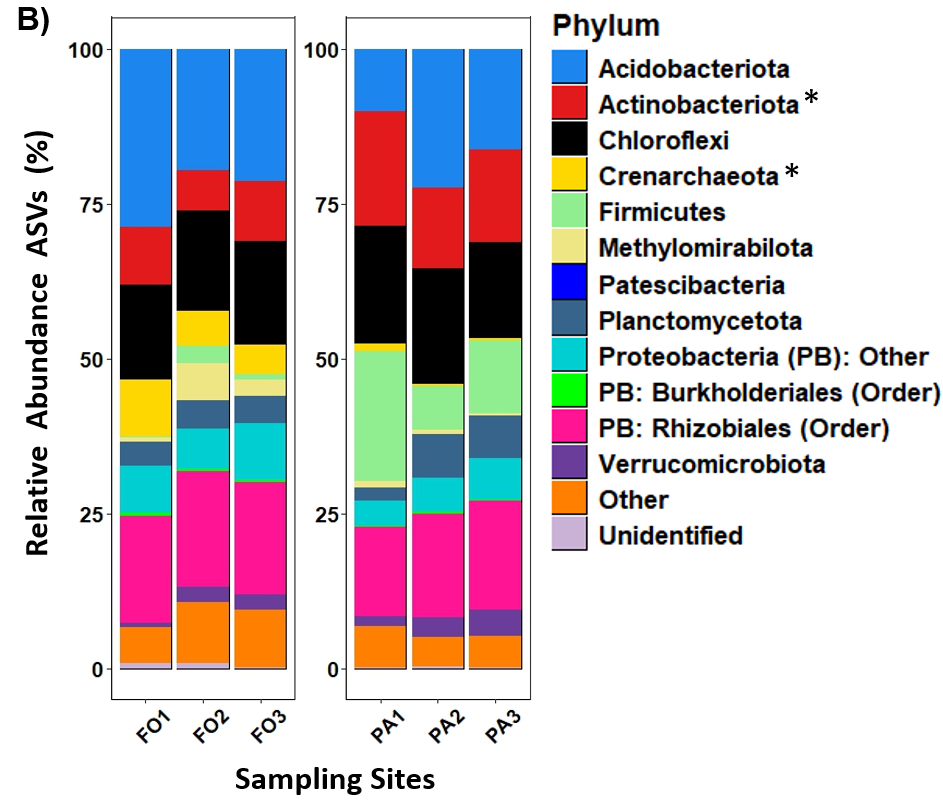


**Fig. S.1.** A)Bray-Curtis dissimilarities of the initial SPC withinforest soils, within pasture soils and between forest and pasture soils. Data was normally distributed (p = 0.739), one-way ANOVA showed no significant difference among the three groups (F_2, 11_ = 1.02, p = 0.39). B) Relative abundance at phylum level of the initial soil prokaryoric community of forest (left) & pasture (right) in each sampling site. “Other” taxon includes 13 phyla with a relative abundance below 1.5%: Armatimonadota, Bacteroidota, Bdellovibrionota, Cyano-bacteria, Desulfobacterota, GAL 5, Gemmatimonadota, Halobacterota, Myxococcota, NB-j, Nitrospirota, RCP2-54 and WPS-2. Asterisks indicate statistically differences between forest and pasture soils: *p < 0.05.

**
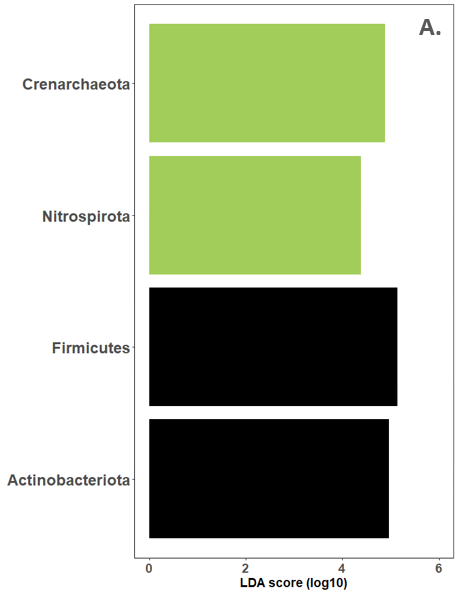

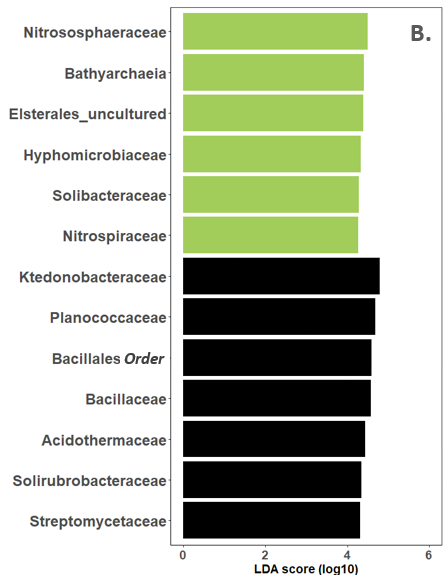
**

**Fig. S.2.** LEfSe analyses of the initial SPC (LDA scores (log 10) > 3.0 and p < 0.05, n=3). (A) Histogram reveals the most differentially abundant phyla between the initial SPC of forest (top green) and pasture (bottom black). (B) Histogram shows the most differentially abundant families between the initial SPC of forest (top) and pasture (bottom).

| **Table S.5 \|** Relative abundances (%) of prokaryotic populations in forest and pasture soils under different drought and root exudate treatments. Different lowercase letters in the same row (forest or pasture soils) mean significant difference based on ANOVA and post-hoc Tukey’s test (F) or Kruskal-Wallis and post-hoc Dunn test (Χ^2^). Different bolded capital letters in initial soils mean significant difference (*) between initial SPC based on Welch t-test. Actinobacteriota (initial soils): t=3.67, p-value=*; Crenarcheota (initial soils): t=-4.2, p-value=*. Asterisks indicate statistically differences: *p < 0.05, **p < 0.01, ***p < 0.001, n.s. (not significant). | | | | | | | | | | | | | | | | | |
| --- | --- | --- | --- | --- | --- | --- | --- | --- | --- | --- | --- | --- | --- | --- | --- | --- | --- |
|  | |  | | **FOREST** | | | | | |  | **PASTURE** | | | | | | |
| **Phyla** | **Initial soils** | | **RD**  **+H_2_O** | | **RD**  **+RE** | **SD**  **+H_2_O** | **SD**  **+RE** | **p-value** | **F/Χ^2^** |  | **Initial soils** | **RD**  **+H_2_O** | **RD**  **+RE** | **SD**  **+H_2_O** | **SD**  **+RE** | **p-value** | **F/Χ^2^** |
| Acidobacteriota | 23.2a | | 14.34a | | 3.86bc | 6.86b | 2.64c | *** | 32.23 (F) |  | 16.27a | 14.2a | 2.16b | 5.77ab | 1.87b | *** | 27.93 (Χ^2^) |
| Actinobacteriota | **8.49bB*** | | 31.92ab | | 38.37a | 27.41ab | 23.27ab | * | 3.66 (F) |  | **15.42A*** | 23.62 | 27.24 | 24.74 | 21.48 | n.s. | - |
| Chloroflexi | 16.13a | | 10.25a | | 2.71b | 3.81ab | 1.59b | *** | 29.19 (Χ^2^) |  | 17.77a | 13.01a | 3.88b | 3.72b | 1.58c | *** | 31.29 (F) |
| Crenarcheota | **6.48aA*** | | 5.24a | | 1.11b | 0.86b | 0.22c | *** | 50.03 (F) |  | **0.62aB*** | 0.69a | 0.2a | 0.05ab | 0.02b | *** | 26.34 (Χ^2^) |
| Firmicutes | 1.33 | | 2.84 | | 1.42 | 6.42 | 3.76 | n.s. | - |  | 13.3a | 13.09a | 4.61b | 11.52a | 10.51a | *** | 7.33 (F) |
| Methylomira-  bilota | 3.07a | | 1.07a | | 0.2ab | 0.3ab | 0.07b | ** | 17.45 (Χ^2^) |  | 0.67a | 0.34ab | 0.14b | 0.08bc | 0.03c | *** | 10.64 (F) |
| Patescibacteria | 0 | | 0 | | 7.32 | 0.02 | 6.14 | n.s. | - |  | 0c | 0.02bc | 3.42a | 1.37ab | 1.36a | *** | 6.315 (F) |
| Planctomycetota | 4.3a | | 2.96a | | 0.77c | 1.57b | 0.34d | *** | 45.94 (F) |  | 5.32a | 3.94a | 1.65ab | 2.1ab | 0.78b | *** | 7.33 (F) |
| Proteobacteria (PB): Others | 7.67b | | 5.28ab | | 3.41bc | 2.27c | 2.08c | *** | 11.10 (F) |  | 5.43 | 5.25 | 2.9 | 2.7 | 2.69 | n.s. | - |
| PB: Burkholderiales (Order) | 0.42c | | 2.16c | | 32.68bc | 41.44ab | 55.57a | *** | 28.41 (Χ^2^) |  | 0.21b | 4.34b | 45.14a | 39.62ab | 56.93a | *** | 24.92 (Χ^2^) |
| PB: Rhizobiales (Order) | 18.07a | | 16.75a | | 5.39b | 6.49b | 2.95c | *** | 39.91 (F) |  | 16.28a | 13.81a | 5.19b | 4.72b | 1.75c | *** | 42.33 (F) |
| Verrucomicro-biota | 1.9a | | 1.52a | | 0.34b | 0.37b | 0.06c | *** | 29.96 (F) |  | 2.96a | 2.39a | 0.79b | 0.33bc | 0.1c | *** | 21.76 (F) |
| Other Phyla | 8.33 | | 5.24 | | 2.27 | 1.88 | 1.12 |  |  |  | 5.51 | 5.03 | 2.52 | 3.12 | 0.86 |  |  |
| Unidentified | 0.62 | | 0.45 | | 0.14 | 0.31 | 0.19 |  |  |  | 0.26 | 0.26 | 0.16 | 0.15 | 0.06 |  |  |
| Total | 100 | | 100 | | 100 | 100 | 100 |  |  |  | 100 | 100 | 100 | 100 | 100 |  |  |
| Initial soils (initial community), RD+H_2_O (using the reference WHC in dry period in Madre de Dios +H_2_O), RD+RE (using the reference WHC in dry period + artificial RE), SD+H_2_O (severe drought +H_2_O) & SD+RE (severe drought + artificial RE). | | | | | | | | | | | | | | | | | |

**
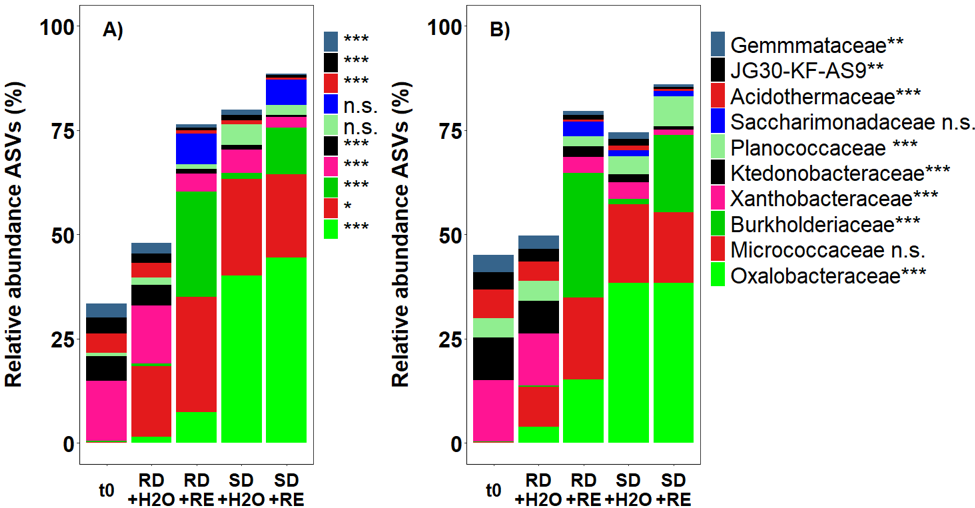
**

**Fig. S.3.** Relative abundances (%) of the top 10 most abundant prokaryotic families under different treatments of forest (a) and pasture soils (b). t0 (initial community), RD+H2O (using the reference WHC in dry period +H2O), RD+RE (using the reference WHC in dry period + artificial RE), SD+H2O (severe drought +H2O) & SD+RE (severe drought + artificial RE). Each family is coloured according to its phylum, as shown in Figure 8. Asterisks indicate statistically differences in forest or pasture soils after the incubation: *p < 0.05, ** p < 0.001, ***p < 0.001, n.s. (not significant).

| **Table S.6 \|** Relative abundances (%) of the top 5 most abundant prokaryotic families in forest and pasture soils under different drought and root exudate treatments. The top 5 families show a relative abundance of more than 10% in one of the treatments. Different letters in the same row (forest or pasture soils) mean significant difference based on ANOVA and post-hoc Tukey’s test (F) or Kruskal-Wallis and post-hoc Dunn test (Χ^2^). Asterisks indicate statistically differences: *p < 0.05, **p < 0.01, ***p < 0.001, n.s. (not significant). | | | | | | | | | | | | | | | | | |
| --- | --- | --- | --- | --- | --- | --- | --- | --- | --- | --- | --- | --- | --- | --- | --- | --- | --- |
|  | |  | | **FOREST** | | | | | |  | **PASTURE** | | | | | | |
| **Family** | **Initial soils** | | **RD**  **+H_2_O** | | **RD**  **+RE** | **SD**  **+H_2_O** | **SD**  **+RE** | **p-value** | **F/Χ^2^** |  | **Initial soils** | **RD**  **+H_2_O** | **RD**  **+RE** | **SD**  **+H_2_O** | **SD**  **+RE** | **p-value** | **F/Χ^2^** |
| Oxalobacteraceae | 0.0b | | 1.5b | | 7.5b | 40.1a | 44.5a | *** | 36.1 (Χ^2^) |  | 0.03c | 4.0c | 15.3bc | 38.4ab | 38.5a | *** | 28.18 (Χ^2^) |
| Micrococcaceae | 0.2b | | 17.0ab | | 27.6a | 23.2ab | 19.9ab | * | 3.14 (F) |  | 0.3 | 9.5 | 19.6 | 18.9 | 16.9 | n.s. | - |
| Burkholderiaceae | 0.4b | | 0.7b | | 25.2a | 1.4b | 11.1ab | *** | 35.36 (Χ^2^) |  | 0.2bc | 0.4c | 29.9a | 1.2bc | 18.5ab | *** | 29.65 (Χ^2^) |
| Xanthobacteraceae | 14.4ab | | 13.9a | | 4.4bc | 5.6abc | 2.6c | *** | 28.5 (Χ^2^) |  | 14.6a | 12.4a | 3.9b | 4.0b | 1.3c | *** | 35.89 (F) |
| Ktedonobacteraceae | 5.9a | | 4.9a | | 1.1a | 1.3ab | 0.5b | *** | 29.9 (Χ^2^) |  | 10.2a | 7.9a | 2.5ab | 1.8ab | 0.8b | *** | 28.65 (Χ^2^) |
| Total | 20.9 | | 37.9 | | 65.8 | 71.5 | 78.6 |  |  |  | 25.3 | 34.1 | 71.0 | 64.3 | 75.9 |  |  |
| Initial soils (initial community), RD+H_2_O (using the reference WHC in dry period in Madre de Dios +H_2_O), RD+RE (using the reference WHC in dry period + artificial RE), SD+H_2_O (severe drought +H_2_O) & SD+RE (severe drought + artificial RE). | | | | | | | | | | | | | | | | | |

| **Table S.7** **\|** Mean emission rates of CO_2_ (μg CO_2_ g^-1^ dry soil d^-1^) at different treatments during the 3 drying-rewetting cycles for both ecosystems (forest and pasture). Each cycle is divided by wet (WHC: 95-50 %) and dry period (WHC: 30-5 %). Mean ± SD. Different lowercase letters in the same row indicate significant differences among treatments. Different bolded capital letters in the same column indicate significant differences among cycles (wet or dry period). Both analyses are based on Kruskal-Wallis & post-hoc Dunn (Χ^2^**)** test or ANOVA & post-hoc Tukey’s test (F). * p < 0.05, ** p < 0.01, *** p < 0.005. | | | | | | | | | | |
| --- | --- | --- | --- | --- | --- | --- | --- | --- | --- | --- |
| **Ecosystems** | | **FOREST** | | | | **PASTURE** | | | |  |
| **RE Treatment**  (N) | | **+H_2_O**  (Wet = 18 / Dry = 12) | | **Artificial Root Exudate**  (Wet = 45 / Dry = 30) | | **+H_2_O**  (Wet = 18 / Dry = 12) | | **Artificial Root Exudate**  (Wet = 45 / Dry = 30) | |  |
| **Drought**  (WHC dry period) | | **Reference**  (30%) | **Severe**  (5%) | **Reference**  (30%) | **Severe**  (5%) | **Reference**  (30%) | **Severe**  (5%) | **Reference**  (30%) | **Severe**  (5%) |  |
| **Wet** | **Cycle 1** | 10.0 ± 4.4 *b* | 8.9 ± 5.6**B** *b* | 85.5 ± 57.8**B** *a* | 87.7 ± 59.3**B** *a* | 10.5 ± 8.12**AB** *b* | 9.7 ± 5.7**B** *b* | 104.6 ± 41.8**B** *a* | 96.9 ± 40.6**B** *a* | *Χ^2^ = 156.7 **** |
|  | **2** | 12.2 ± 7.5 *b* | 33.1 ± 22.4**A** *b* | 155.8 ± 103**A** *a* | 191.4 ± 113.1**A** *a* | 28.2 ± 47.8**A** *b* | 31.4 ± 18.0**A** *b* | 162.7 ± 6**A** *a* | 185.3 ± 81.3**A** *a* | *Χ^2^ = 140.1 **** |
|  | **3** | 10.3 ± 4.1 *b* | 21.0 ± 9.8**A** *b* | 177.4 ± 130.6**A** *a* | 184.4 ± 117.9**A** *a* | 8.3 ± 4.34**B** *b* | 17.5 ± 12.6**B** *b* | 172.9 ± 69.4**A** *a* | 184.2 ± 72.7**A** *a* | *Χ^2^ = 157.1 **** |
|  |  | **n.s.** | **F = 20.68 ***** | **Χ^2^ = 20.54 ***** | **F = 22.51 ***** | **Χ^2^ = 6.62 *** | **F = 15.77 ***** | **F = 17.61 ***** | **F = 29.2 ***** |  |
| **Dry** | **Cycle 1** | 3.3 ± 1.5**A** *ab* | 1.0 ± 0.3 *bcd* | 13.3 ± 12.9 *a* | 0.9 ± 0.5 *cd* | 3.4 ± 2.5 *abc* | 0.4 ± 0.3 *d* | 28.7 ± 30.2 *a* | 0.5 ± 0.5**B** *d* | *Χ^2^ = 135.6 **** |
|  | **2** | 2.7 ± 0.7**AB** *ab* | 0.9 ± 0.4 *bcd* | 16.2 ± 11.7 *a* | 1.1 ± 0.5 *bcd* | 2.1 ± 0.5 *bc* | 0.5 ± 0.3 *d* | 15.9 ± 10.2 *a* | 0.9 ± 0.8**AB** *cd* | *Χ^2^ = 136.9 **** |
|  | **3** | 2.3 ± 1.2**B** *bc* | 0.9 ± 0.2 *cd* | 10.7 ± 6.6*ab* | 1.0 ± 0.6 *cd* | 1.9 ± 0.7*bc* | 0.4 ± 0.3 *d* | 19.0 ± 11.0 *a* | 1.1 ± 0.8**A** *cd* | *Χ^2^ = 133.5 **** |
|  |  | **F = 4.2 *** | **n.s.** | **n.s.** | **n.s.** | **n.s.** | **n.s.** | **n.s.** | **Χ^2^ = 4.67 *** |  |

| **Table S.8 \|** Carbon mass balance of the incubation experiment. Carbon (C) values are presented in mg C per incubation unit. Each incubation unit contained an equivalent to 10 g of dried soil. Unbalanced net C might be caused by the heterogeneity of the initial C in the soils, measurement inaccuracies, minor C losses during flushing with artificial air or by changes due to unmeasured C microbial biomass, or microbial transformations such as CH_4_ and other volatile organic compounds emissions not specifically measured. | | | | | | | |
| --- | --- | --- | --- | --- | --- | --- | --- |
| **Treatment** | **N** | **Initial C content**  **in the soil**  (endogenous + RE solution) | **Cumulative**  **C-CO_2_ emitted** | **C-CO_2_ emitted (%) from the initial C content** | **Final C content**  **in the soil** | **Final C in the soil +**  **cum. C-CO_2_ emitted** | **Net C balance (%)** |
| **Reference Drought** |  |  |  |  |  |  |  |
| Forest +H_2_O | 6 | 96.2 ± 22.5 | 0.64 ± 0.4 | 0.7 ± 0.3 | 95.8 ± 22.6 | 96.5 ± 22.8 | + 0.6 |
| Pasture +H_2_O | 6 | 82.2 ± 16.9 | 0.79 ± 0.7 | 0.9 ± 0.8 | 72.0 ± 14.8 | 72.8 ± 15.4 | - 10.2 |
| Forest Root Exudate | 15 | 120.2 ± 21.5 | 4.46 ± 0.5 | 3.8 ± 0.8 | 99.6 ± 19.2 | 104.1 ± 19.0 | - 13.3 |
| Pasture Root Exudate | 15 | 106.2 ± 16.0 | 5.20 ± 0.7 | 5.1 ± 1.3 | 78.4 ± 14.1 | 83.6 ± 13.5 | - 20.9 |
| **Severe Drought** |  |  |  |  |  |  |  |
| Forest +H_2_O | 6 | 96.2 ± 22.5 | 0.59 ± 0.2 | 0.7 ± 0.3 | 98.6 ± 27.3 | 99.2 ± 27.2 | + 2.5 |
| Pasture +H_2_O | 6 | 82.2 ± 16.9 | 0.56 ± 0.1 | 0.7 ± 0.2 | 72.1 ± 11.9 | 72.6 ± 12.0 | - 9.8 |
| Forest Root Exudate | 15 | 120.2 ± 21.5 | 4.40 ± 0.6 | 3.7 ± 0.6 | 102.3 ± 18.8 | 106.7 ± 19.0 | - 11.1 |
| Pasture Root Exudate | 14 | 106.2 ± 16.0 | 4.58 ± 0.5 | 4.3 ± 0.5 | 87.0 ± 12.5 | 87.4 ± 12.6 | - 14.5 |

* *

| **Table S.9** **\|** Mean α-diversity indices (non-parametric Simpson diversity index and observed amplicon sequence variants) at different treatments following the incubation experiment (mean ± standard deviation). Different letters in the same column mean significant difference based on ANOVA and post-hoc Tukey’s test (F) or Kruskal-Wallis and post-hoc Dunn test (Χ^2^); p-value < 0.05. | | | |
| --- | --- | --- | --- |
| **Treatment** | **N** | **Simpson** | **Observed ASVs** |
| **Initial soils** |  |  |  |
| Forest | 3 | 0.992 ± 0.001a | 524 ± 80abc |
| Pasture | 3 | 0.991 ± 0.001ab | 589 ± 78ab |
| **Reference Drought** |  |  |  |
| Forest +H_2_O | 6 | 0.979 ± 0.01a | 547 ± 32ab |
| Pasture +H_2_O | 6 | 0.986 ± 0.01a | 637 ± 51a |
| Forest Root Exudate | 15 | 0.922 ± 0.05abc | 498 ± 38b |
| Pasture Root Exudate | 14 | 0.935 ± 0.02abc | 445 ± 83bcd |
| **Severe Drought** |  |  |  |
| Forest +H_2_O | 6 | 0.862 ± 0.04c | 440 ± 78bcd |
| Pasture +H_2_O | 6 | 0.918 ± 0.03abc | 483 ± 109bc |
| Forest Root Exudate | 14 | 0.871 ± 0.06c | 354 ± 73d |
| Pasture Root Exudate | 14 | 0.908 ± 0.03bc | 381 ± 105cd |
|  | | Χ^2^ = 54.592 | F = 10.77 |

* *


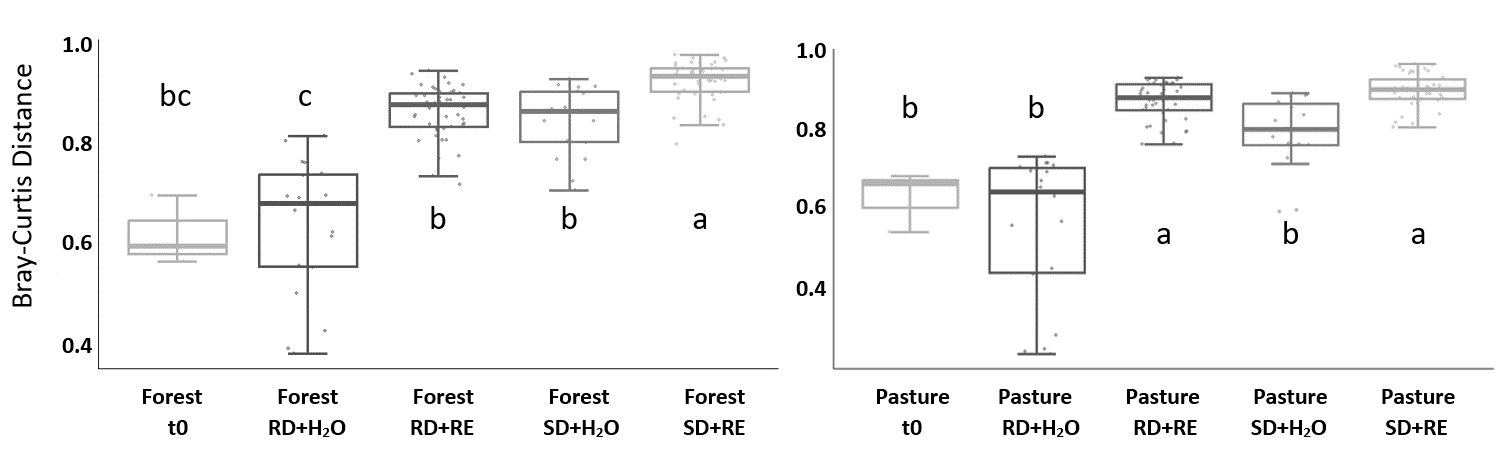


**Fig. S.4.** Boxplots illustrating the Bray-Curtis dissimilarity indices between the initial SPC (t0) and the communities after the treatments in forest (A) and pasture soils (B). t0 represents the natural variability within the initial community, RD+H_2_O (using the reference WHC in dry period in Madre de Dios +H_2_O), RD+RE (using the reference WHC in dry period + artificial RE), SD+H_2_O (severe drought +H_2_O) & SD+RE (severe drought + artificial RE). Data was not normally distributed, different letters mean significant differences based on Kruskal-Wallis and post-hoc Dunn test, p < 0.05.

| **Table S.10 \|** Pairwise PERMANOVA results for between-treatments differences in soil prokaryotic community composition. RD: reference WHC during the dry season in the study region; SD: severe drought; +H_2_O: only water was added, +RE: root exudate addition. | | | | | | | |
| --- | --- | --- | --- | --- | --- | --- | --- |
|  | +H_2_O | | |  | +RE | | |
| **Pairwise PERMANOVA** | *F_1_* | *R^2^* | adjusted *p* |  | *F_1_* | *R^2^* | adjusted *p* |
| Forest RD vs Forest SD | 6.260 | 0.385 | **0.006** |  | 7.078 | 0.208 | **0.006** |
| Forest RD vs Pasture RD | 3.179 | 0.241 | 0.06 |  | 7.221 | 0.211 | **0.006** |
| Forest RD vs Pasture SD | 7.124 | 0.416 | **0.024** |  | 13.761 | 0.338 | **0.006** |
| Forest SD vs Pasture RD | 7.520 | 0.429 | **0.012** |  | 9.352 | 0.265 | **0.006** |
| Forest SD vs Pasture SD | 2.323 | 0.188 | 0.342 |  | 10.148 | 0.281 | **0.006** |
| Pasture RD vs Pasture SD | 4.050 | 0.288 | **0.048** |  | 4.284 | 0.142 | 0.054 |
